# Supplementary material for: Post-traumatic growth in mental health recovery: qualitative study of narratives
Source: BMJ Open. 2019 Jun 28;9(6):e029342. doi: 10.1136/bmjopen-2019-029342 (PMC6609070; doi:10.1136/bmjopen-2019-029342)
Supplement: Supplementary file 1 [file bmjopen-2019-029342supp001.pdf]

Online Supplement 1

Guidelines for promotional material

All promotional material produced to support recruitment in NEON Phase 1, for both activities 1 and 2, will incorporate the following:
1. A summary of the objectives of the project as a whole, e.g.
"The NEON Study looks at whether experiencing online stories of personal recovery ('recovery narratives') told by peers improves quality of life for people with psychosis."

2. A summary of the objectives of phase 1, e.g.
"The aim of phase one is to develop a theoretical understanding of how recovery narratives (personal stories of mental health problems and recovery) can be of benefit to other people with mental health problems."

3. A summary of the group which the promotional material is aiming to recruit, e.g.
"We are seeking to recruit 30 participants who are working in statutory or voluntary roles where having lived experience of mental health problems is a requirements, and where sharing personal experiences is a normal part of the work of the role"

4. A summary of what participation would require, e.g.
"Each participant would engage in a single one-hour interview, where they will be asked to talk about their experience of recovery".

5. The corporate identity of Nottinghamshire Healthcare NHS Foundation Trust (NHCT), as the project sponsor (e.g. see header of this document)

6. If this piece of recruitment is located in a site other than NHCT, then the corporate identity of the site where it is being conducted.
This will be either South London and Maudsley NHS Foundation Trust (SLAM) or East London NHS Foundation Trust (ELFT).

7. A mechanism to allow people to find out more information, e.g.
- An email address or telephone number
- An information page on the project website

8. Information about the funder and mechanism, e.g.
"This project has been funded by the National Institute of Health Research, as a Programme Grant for Applied Research (PGfAR)"

9. Information about approvals that have been received, e.g.
"This study has been reviewed and given favourable opinion by INSERT REC INFO and CONTACT INFO. The project has also been reviewed by the Health Research Authority, and by the Research and Innovation Team at Nottingham Healthcare NHS Foundation Trust, who are sponsoring the project."

Online Supplement 2

Preliminary coding framework for post-traumatic growth in psychosis and other severe mental health conditions

#	Code	Definition	Examples
1	Strengths	New or improved use of personal strengths or resources	More resilience, confidence, self-efficacy, skills at navigating life challenges, inner strength, determination
2	Relationships	Improved relationships with others	Better connection with friends or family, new meaningful relationships
3	Purpose	New possibilities and purpose in life	Fresh directions, e.g. career, leisure pursuits; finding a place or role in society
4	Life appreciation	Greater or altered appreciation of life	Finding new meaning in experiences, seizing opportunities, finding new life purpose or direction
5	Spirituality	More engagement with spirituality or existential questions	New spiritual practice, (re-)connecting with an organised religion, deeper life meaning
6	Character	Developing positive character traits	More creativity, compassion for others
7	Lifestyle	Making positive lifestyle changes	Better sleep, more activity, healthier relationship with food / drink, developing new interests, choosing healthier relationships
8	Identity integration	Integration of experience of psychosis into identity	Psychosis experiences as shaping who the person is now, psychosis being part but not all of identity, more insight or clarity
9	Self-acceptance	More self-acceptance and awareness	More self-compassion, positive sense of self
10	Other	Other forms of post-traumatic growth not coded above	

Online Supplement 3

NB The interview was in two parts. In Part 1 the participant was asked to tell their story of recovery. In Part 2, follow-up questions were asked about the impact of their stories on others. The topic guide for Part 1 did not change, and only data from Part 1 (i.e. the participant's open narrative) are reported in the current study.

Interview topic guide

START OF TOPIC GUIDE

Preparation

Before the participant arrives, allocate a unique identifier (UID) to the participant, and write it into a blank Informed Consent Form.

Introduction

- Introduce the interviewer facilitator
- Ensure the participant has read the information sheet and understands that participation is voluntary and that they are free to withdraw at any time
- Explain the aims and purpose of the activity and give a brief description of the interview structure. Tell the participant they can decide whether their story is used in the later part of the NEON study, emphasising that this may mean other people beyond the study team may see it.
- Provide an opportunity for participant to ask any questions, and then obtain written consent through the Informed Consent Form
- Describe digital recording of the interview. Clarify whether the participant wants to be video-recorded or audio-recorded.
- Turn on the recorder. Read out the UID so that it is recorded.

Questions

Part 1

Ask the participant to describe their own story of recovery. Do not use language (e.g. 'psychosis') which might be leading – refer to e.g. 'mental health difficulties'. Give them plenty of time, listen carefully. Reply if asked (e.g. "Is this okay?" – "Yes, you're doing great") and use minimal prompts if needed (e.g. "Do go on") but try to let the participant tell their story in their own words.

Part 2

Follow up with questions about the narrative, e.g.

1. Who have you shared your story with, and why?
2. What was the impact on the recipient and on you? If there was a particularly powerful part of their narrative, ask specifically about the impact of that part of their story.
4. Do you sometimes hold back some aspects of your story? If so, how do you decide what and when to hold back?

3. Has anyone else shared a recovery narrative with you? What parts of someone else's story made an impact? Why do you think that part made an impact?
4. How has the way you have told your story changed over time? Why do you think this is?

If the participant becomes distressed during the interview, ask if they would like to take a break or stop. If the participant reveals information which is of concern and may need reporting i.e. potential risks to another person or to themselves, or criminal behaviour, then continue the interview if you feel comfortable to, but discuss these with the PI at the earliest opportunity and where appropriate report accordingly.

End of interview
Explain the interview is now over and ask if the participant has any final questions. Give information about timeline for the study and how publications can be accessed. Thank the participant for their participation.

END OF TOPIC GUIDE

Online Supplement 4

Interim coding framework for post-traumatic growth in psychosis and other severe mental health conditions

#	Code	Definition	Examples
1	Strengths	New or improved recognition or use of personal strengths or resources	More resilience, confidence, self-efficacy, skills at navigating life challenges, inner strength, determination, positive character traits
2	Relationships	Improved relationships with others	Better connection with friends or family, new meaningful relationships, moving away from unhealthy relationships
3	Purposefulness	New possibilities and purpose in life	Fresh directions, e.g. career, leisure pursuits; finding a place or role in society, social or political activism.
4	Life appreciation	Greater or altered appreciation of or reflection on life	Fresh perspectives, new meaning-making framework. Finding new meaning in experiences, seizing opportunities, finding new life purpose or direction, new appreciation for informal or formal learning, new or different education or career development
5	Spirituality	More engagement with spirituality or existential questions	New spiritual practice, (re-)connecting with an organised religion, deeper life meaning
6	Self-discovery	Having a changed, fuller and deeper understanding of oneself and how to live in the world	More creativity, compassion for others, increased empathy for others, new skills
7	Lifestyle	Making positive lifestyle changes to maintain wellbeing and support self-care	Better sleep, more activity, healthier relationship with food / drink, developing new interests, choosing healthier relationships, changing social environment
8	Identity integration	Integration and sense-making of experience of mental health issues into identity	Mental health experiences as shaping who the person is now, psychosis or other mental health experiences being part but not all of identity, more insight or clarity, re-framing experiences, choosing other explanatory frameworks to make sense of experiences
9	Self-acceptance	More self-acceptance and self-compassion	More self-compassion, positive sense of self, less internalised stigma
10	Other	Other forms of post-traumatic growth not coded above	

Online Supplement 5

Final conceptual framework for post-traumatic growth in psychosis and other severe mental health conditions

Type of growth	Definition of the positively perceived change	Illustrative quotes
**1. Self-discovery**	**Having a fuller and deeper understanding of oneself**	
1.1 Emotional life	Discovering or re-discovering how to access, accept and be mindful of inner emotional life and difficult feelings	But what I will say is that, er, I found that the, my, what was going on was an internal not an external thing. Because before that I, I've moved about nine times, I'd move, I'd just move somewhere else and thought it's going to be different now. At one point I moved to the States, you know? And I'd be in the same situation in the States that I'd be in England. (B04)<br><br>There was just a point where I was just like I forgot how to feel, I forgot how to be hungry, I forgot how to feel tired, I forgot how to be exhausted, I forgot how to feel fear and then I realised oh my fuck I need to feel fear, you know, I need these things, these things are really fucking important, you know that a human being needs fear, we need tiredness, we need hunger…I moved to Hong Kong and it found me again and I realised it wasn't really my situation, it was me, it was something inside me that was drawing this energy towards me and there I, unless I changed the noise inside, the noise outside is never going to go away. (B25)<br><br>And I think that is the key to everything isn't it, accessing your emotions, not running away from them, which a lot of people in mental suffering with mental health are trying to do because it is overwhelming and painful. So it is about yeah feeling and not feeling somebody else's or not, following somebody else and not being scared to be who you are and be different I think. (C19)
1.2 Self-	Knowing oneself	I feel like I know myself quite well, you know I can heal myself. And uh, these are things

knowledge	better, being more authentic and not being as shaped by the expectations of others	that people actually take for granted but took me a very long time to connect to how are you feeling you know and what do you want right now? (A08)<br><br>I think you find out who you are and sort of how you have to live to keep yourself sane in a way. (A14)<br><br>When I discovered that freedom, that I didn't have to join the rat-race of having a car, wife and three kids, you know, having a job, that was quite liberating… I was constrained by what people wanted me to do, instead of what I wanted to do. (B18)<br><br>People or old friends that would say…you've got to stop being a bit like this, actually I would take that and think to myself am I? Am I a bit like that? And question what they've said and then if I thought well no actually I'm not like that anymore, you know, then I've turned around and said well actually, you know, that's wrong, you know, because that's, that's, that's not me and you can't tell me I am something if that's not what I am. You know, well you can but you know I'm not gonna listen to you. And uh you know by, by questioning uh what people was saying, uh sort of, uh, removed that vulnerability I think, and um, by removing vulnerability has allowed me to feel more in control of myself and um, who I am. (C03)
1.3 Self-acceptance	Grieving and letting go of the past, and developing self-compassion	I grieve all the hopes that are not there, you know things that haven't worked out, and I try to accept what is there and say well this is it, this is the thing that is real, maybe the other things were a little fantastical… And so I still talk to myself a lot but it's not now, it's, it's, it's me comforting myself and actually communicating with me, you know, just sort of, um it's that thing of having learned to love myself. (A08)<br><br>I started going to loads of classical music concerts which I'd really shunned because I felt like I'd really failed as I'm not playing the violin anymore, I should be, I've really failed, I'm not good as the other people who have made it their profession. Suddenly I was like, God that doesn't matter, that is just so irrelevant, like classical music and music generally is so beautiful I just want to go and listen to it. (A10)<br><br>It's all about self-accepting, getting to know me and it helped. (A17)

		I feel like I'm at peace with a lot of the, erm, depression that I felt over things that have happened in the past. (B01)<br><br>The key word is accepting the situation that I was in, um, and being honest with myself. (C03)
1.4 Self-responsibility	Taking (back) responsibility for one's own life	Yeah, because in my own mindset and recovery…I had to become interested in the problem because prior to that my outlook on recovery in mental health was based on seeing a Consultant Psychiatrist and they will have the answer because that is the predominant feeling, patient/doctor relationship, you see a doctor, you trust what they say and then I had to become interested after I realised that the doctors can't fix me. So when I became interested I became open to learning. (A21)<br><br>The one thing he did when he came, when he saw, he said, 'I'm not going to help you do one thing in this flat unless you start doing it for yourself. If you make a start yourself, I will help you. If you don't do anything, I'm gone. And that hit me like a, er, thunderbolt, knowing that, knowing I, that if I'm not going to help myself, no one else will help me. And that was the beginning, really, of my recovery. (B02)<br><br>Everything I do is like an attempt to rebuild my life but it's taken a long time. (B05)<br><br>Maybe sometimes we need to go through that process, in order to think, I don't need to go back there, to where I was ten years ago, thinking about that experience anymore. But you can think about it, but have a different sort of attitude to it. (B06)<br><br>I tend to be living the moment, that's part of my new self, this post-near death experience self is to really be living in the moment as much as possible, not worry about the future or churn over the past. (D04)<br><br>This was like taking a step back and looking at almost re-engineering life to take into account self-care, self-preservation and also building myself up rather than saying rotten stuff about myself and focusing on what I couldn't do and what I should do and should be

		able to do and all of that. (D08)
**2. Sense of self**	**Development of a more positive sense of self, including integration and valuing of illness experiences**	
2.1 Pride in self	Taking pride in oneself, including personal strengths and achievements	I feel quite proud of my stability. (A01)<br><br>I'm proud of that and that makes me feel better and enables me to operate…I've worked on myself throughout my life and I'm quite proud of that. (A12)<br><br>I believe in my self-worth these days…I must pat myself on the back. (A15)<br><br>Doing well, embracing who you are, loving who you are. Because I went through a long period of neglecting myself, my identity, my race, my culture. (B08)<br><br>I think that's something I'm pretty proud of actually, that I just take people as they are. (B21)<br><br>So yeah, I'm proud of where I've come, do you know what I mean, how far I've come, and that's what help, holds me from stopping and falling apart completely when I think about my daughter, and I get real mad sometimes…That knowing what I've got, and how I've achieved, and I don't want to lose it, cos when she comes to find me I want her to see that I've got these things, and that I'm not a complete arsehole living in a hostel, know what I mean? (C10)
2.2 Integration of experiences	Illness experiences become an accepted part of one's sense of self	That stuff has happened in my life and that is just how it has turned out. Um yeah, that is unfortunate but it is not going to define everything about me. (A07)<br><br>If there was a big red button on the table and somebody said would you like to switch off your diagnosis I would say no because I have accepted it now. Although it was horrid, it

		was horrible, I can't take anything away from that, but I have accepted that it has become part of my personality and I am happy with that and that's me. (A21)<br><br>I think for a long time I was just existing with the mental health, getting to know, getting to understand it…Because there's very much negativity around it and I felt ashamed. (B06)<br><br>You don't choose the issues that you've got but you can, you can make a choice to change. (C04)<br><br>I am who I am because of what happened. (D04)<br><br>The more I was able to make sense through telling my story of what had happened to me, it then started to have a meaning. (D11)
2.3 Valuing of experiences	Finding positives in the experience of illness	I've had all these amazing experiences and you are just saying it's illness, I just didn't believe it…I feel that it's important and the messages I've got from being in an alternative reality have been extremely important. (A10)<br><br>What they were calling symptoms that must be eradicated, were actually part of me and so I looked behind that and said that is where, that is where my creativity comes from. This is where, this is how I am able to take a photograph, this is how I am able, when I am sitting at a piano to think of a tune. (A19)<br><br>Whereas before I felt ashamed to have these mental health issues, I now feel really proud in a way because I know that I can use it for good and I can make a difference in my community. (B01)<br><br>There is a liberation that comes through all of this, a personal kind of yeah, not sure it's full blown self-actualisation, but it's somewhere up close to the top of the pyramid. (D04)<br><br>I am still me but I am a different me and I am stronger. (D05)<br><br>I kind of thought that that was the best I could ever hope for and that was fine because I

		kind of really internalized my label of I am an anorexic, I am a you know a broken person who needs a lot of support and actually it was seeing the people who were talking about their lived experience with lots and lots of confidence and taking lots of responsibility and um being empowered to, to self-manage and use their difficulties in a really positive way which kind of opened my eyes to like gosh maybe I am more than my label, maybe I am, maybe I could do more than just function or survive, maybe I could thrive. (D014)
**3. Life perspective**	**New or renewed appreciation of or gratitude about aspects of life**	
3.1 Appreciation of life	Appreciation for life and the importance of hopefulness	I suppose I've become one of those ridiculously, someone I thought I'd never be, I'm becoming one of those ridiculously ever hopeful, ever optimistic people who say there is hope, my life is a life that is about hope, I hope. (A08)<br><br>I have kind of realised that I am not in a position to dictate the terms of life: “it should be like this and I expect this and why is it not like this”. Just, I am alive, I appreciate that I am alive and that I have got so many hours each day to gain something from, some joy or some inspiration or blessings and gifts everywhere that I can and the number one thing is to just keep going, I am a mother and I have to keep going. (C19)
3.2 Appreciation of support	Gratitude for support received from services	So it [organisation] completely changed my view of life. And it's a, it's a roller coaster, it's not like every day's joyous, things would dip, challenges still come in but I deal with them completely differently. Yeah it's like I've found happiness that I never knew existed. (A11)<br><br>Rehab and coming to <service name 1> changed my life, it's like <worker> got me on a college course, it has been absolutely wonderful, it really has, I did a theatre group … and through the rehab I got told about [inaudible] and I came down here <service name 2> and this place is amazing, the things they do here for anybody involved in sex work is absolutely amazing. (C18)
3.3 Meaningful suffering	Gratitude that suffering was meaningful and not in vain	Wow I see where I am and I go back to then and I just think 'you didn't go through that in vain'. (A17)<br><br>So I think I'm, I suppose I am grateful, for want of another way of putting it, that I have

		lived the life I have, I have had these experiences. (D04)<br><br>And it's okay, because I wouldn't be where I was, had I not had the experiences that I've had. I don't know what sort of person I'd be, but I certainly wouldn't be where I, doing what I'm doing now. (D12)
3.4 Survivor mission	New growth of political consciousness or use of illness experiences to benefit others	<Organisation> is about re-framing mental illness as a possible catalyst for possible transformation, so and the aim is to give hope to as many people as possible through the power of story-sharing, so I'm really grateful and passionate about this. (A11)<br><br>I just see myself as hopefully being a beacon to others who are, you know, struggling, and others who are finding things difficult. And they can, you know, refer to me [inaudible] support person and they can look at me and think ‘He can make it through all the things that happened to him, then I can make it too’. (B02)<br><br>I think it’s really important to give back to society. And I think that’s what I’m doing through my work. This new chapter I’m going to start, with women now. (B06)<br><br>And so, you know, I felt like I was becoming something. Erm, I could identify with more, kind of, politicised…the personal was political and I was beginning to become aware of that on a deeper level. So, I thought right, that’s it and, I’m going to set up my own thing. (B09)<br><br>And then as I came back out it was just like no, I wanted to help, I wanted to do something, I wanted to use this story, I wanted to actually like you know... I wanted, that's when I started studying psychology and then I applied to go study in <place>…what's helped me recover was I find life, you know, I've got stuff I want to do, I want to help people, I want to, I want to do psychology. (B25)<br><br>I've been looking forward to sharing my experience because um, by people sharing theirs has really helped me and you know, and that makes it feel like talking about it is a really worthwhile thing to do, because its gonna allow somebody else to grow, or just take a little bit from it that you know, it might benefit them or it might not, maybe they

		need to hear a different story but, you know, somebody might, you know, might hear it and think well I'm really pleased that I've heard that because that's, that's gonna help me. (C03)<br><br>I guess in my recovery I tried to be that person for other people, the thing that I didn't have. (C04)<br><br>And my journey through prostitution, uh so I can talk to people about it now. And that's what I want to do, I want to go on and help people if I can that have been through the same thing. (C18)
**4. Wellbeing**	**More active engagement in, and management of, one's own wellbeing and lifestyle**	
4.1 Motivation	Increased determined to stay well, self-manage and not return to a bad situation	The violence started again and I had two broken jaws, a broken nose, scars on my head and scars on my body, but the biggest scar was in my heart and in my mind. I ended up... being put into a secure unit this time and I was meant to be there for three months. It ended up being two weeks, becoming a voluntary patient for a year. Touch wood and whistle, I swore from that day on, no man, money, love or beast would ever put me back into that situation again and I have stuck to it. (A15)<br><br>I started to take my medication, I got rid of this awful man in my life who was my daughter's father. Which was very liberating and, suddenly had an epiphany through all the craziness and thought, I don't want this in my life anymore. If I get rid of certain things it will decrease some of the stress. (B06)<br><br>I have been that low. And that ill, I have been there and I have seen the light and…I have come back from it. I have actually come out of it, I've been there, right there to the lowest of the low. And pulled myself back from it. And that's difficult to do. (C12)

		When I came out of hospital I was like “I can’t go back there again so I’ve got to sort myself out and I’ve got to build a better relationship with me and the girls”, you know, they’d been through so much. (D05)<br><br>Rather than just aimlessly sort of treading water and doing things that I felt I was sort of having to do or other people were you know were saying I should do you know sort of planning about things that actually I enjoyed. (D13)
4.2 Being active	More engagement in the arts, music, sport, nature and learning	I think the art has given me great kind of, great kind of structure. (A01)<br><br>I didn't go into that dangerous space again but I did meet a lot of like-minded people and I played my violin a lot and I decided I wanted to do something with my violin…I would get like a kind of vision of what my purpose was, what my true purpose was in my life and, it was like you know, playing the violin, teaching the violin, it was, which I hadn't been doing because I was working in admin but it just felt really strongly, that is what, it wasn't to do with competition it just felt like, that's the right direction for me to go in and I need to go more and more in that direction, it felt like, like it was connecting me to my soul purpose somehow. (A10)<br><br>I am someone that struggles with relaxing. So, um, gym, I find that I am someone who has to go to the gym, I have to exercise, um and as ridiculous as it sounds, eating as well. You know like I am someone that needs to treat myself good. In order to be good in myself. (A14)<br><br>So that was quite liberating and I didn’t think that it would help my mental health without even realising, but I think it did, I did become a bit obsessed with jogging because it was something, I guess, a bit of a break from my children and the monotony of life and the difficult relationship I was in, and the difficulties I had. (B06)<br><br>And erm, and I believe that I need to give more time, a bit more time to, to interests. You know, like, er, I love nature. Absolutely adore nature, it’s one of the places I’ve always felt safe, I could go and sleep in forests, more than I could go and sleep in someone’s house. (B09)

		I've been reading quite a lot and educating myself on schizo-affective disorder, plus all the things I've learned about meditation and that kind of thing I practice daily, and I've just found learning to be so rewarding and things, learning through recovery college as well, even though they do suggest reading up on your condition and that kind of thing. So I've just found learning to be so therapeutic and rewarding that I feel like I am at a point now where I can actually study and put my mind towards…doing something worthwhile. (B10)<br><br>I play a lot of music, music is like my, it's my salvation. (B25)<br><br>I could see my body changing and I felt stronger and I felt better and I stopped drinking for a month and I just felt amazing! (D05)<br><br>It was also about sort of about finding activities that I enjoyed much more, rather than just aimlessly sort of treading water and doing things that I felt I was sort of having to do or other people were you know were saying I should do, you know, sort of planning about things that actually I enjoyed… One of the things I did was I joined the choir and you know it's one of [inaudible] gospel choir in South London for about, ever since I got there really and it's a very, very supportive environment, so that's been really critical and I've been able to sort of go there when I've been in every kind of mood state possible. (D13)
**5. Relationships**	**More actively choosing and valuing relationships with others**	
5.1 Choosing relationships	Actively choosing relationships to continue, to re-start or to end	He (counsellor) used a waiter analogy where the waiter is trying to please the clients but then more and more people come in and he is still trying to please them all but the quality of the food comes down and then people start complaining and then it is mad, so basically just concentrate on what you can do and don't try to please everybody…I also now try to mix with people who are just happy and positive and try to stay away from

		people who aren't. (A09)<br><br>But you need the right quality of people around you, network, for you to be able to stay in your mental health, going forward. (B02)<br><br>I got rid of this awful man in my life. (B06)<br><br>I needed to go back to a couple of my old primary school friends' houses and ask for their forgiveness for something. (B07)<br><br>I'd also come to realise before then that the relationship I was in was not quite what it was as he told me lots of things that were untrue that I discovered so I ended that as well and that was having a clean slate but it took me a long time to get literally over it. (B17)
5.2 Valuing relationships	Placing more value on relationships with others	My children they come at the weekend I give them 100%. I give them 100% because I want to be giving them 100%. (A01)<br><br>But then in another sense it's brought the incredible closeness with people with mental health with my, with my immediate family and friends so it has different angles. (A03)<br><br>It's been a process of learning that I needed, I need desperately, I desperately needed family, you know, people I feel safe with, to be myself. And I needed people to help me normalise my feelings rather than be freaked out and hiding and terrified of expressing. (B09)<br><br>Now I do feel a lot more stable than I did back then, and then when I go to these kinds of experiences you know, I've started to like, connections have become my thing because connections are what saved me from like, connections with people, a spiritual connection and this connection between people, there's interconnection between people is essentially what saves people…That power of coming together is so powerful, that is worth fighting for, worth dying for, it's worth me giving my time for. (B25)<br><br>Having the friendship, the respect that built up over that in learning from each other, you

		know educated, talented, intelligent people. (C04)
5.3 Empathy	Enhanced ability to empathise with others	I can empathise and I do empathise and I use empathy in my work a lot. (A12)<br><br>So my purpose really is young people and even when I see the destruction that young people are going through I never blame them, I said it stems from somewhere…I knew what I went through as a young girl. I could have been a prostitute, I could have been on drugs, I didn't. Do you see what I'm saying? So therefore it's deep. (A17)<br><br>You have understanding, empathy…You really empathise. (B15)
**6. Spirituality**	**Deeper engagement with spirituality, religious and existential endeavours**	
6.1 Spiritual awareness	Increased awareness of the presence of something greater than oneself making a positive contribution by providing meaning	Although the experiences were horrendous, and they were horrendous, there's no other way of describing the agony, it has been necessary because I couldn't do it on my own, so it still feels, and maybe this is just you know the spiritual aspect of my life but it still feels as though I do have like something watches over me and is helping me um and I survived. (A08)<br><br>I kind of relived stuff and at the same time just physically I wanted to let go of loads of things, I just kind of clutter cleared my whole house, really cleaned it, I got rid of loads of bits and pieces that I didn't need any more and as I went through that whole process it was like this massive opening, just kind of spiritual opening again…I felt very very free, I felt like I was being guided with everything, I felt very, very spiritual. Um lots of things happened that were very healing I suppose, played a lot of music…when I relived it and the emotion that was associated my voice really did, like it changed, it kind of opened out and it became much more free and liberated and um at the same time I felt like I went into a completely different reality, it was really incredible, it was like intensely spiritual and a feeling that everything was connected and it was though lots of chakras opened or something happened in my body and my mind. (A10)

		Now I'm sort of growing older I know they're spirit animals, I still have them and so it's the wolves that are the most powerful so I do feel protected by those. Um and the other one is [inaudible] spiritual as well I always used to feel some [inaudible] egocentric, sort of like, wounds, [inaudible] kind of reaching out to people because I've always felt like I'm [inaudible] ever since I was really little which has why I have gone into mental health, to help people. (A18)<br><br>But I think the longer deal is this, this, internal shift, they call it a psychic shift, an internal psychic shift, which is more about that spiritual experience and I don't know anything else that, that would work with me, do you know what I mean. And so, erm, since about 2009, that's what I've been kind of exploring in a, in a sort of ad hoc way. (B04)<br><br>But when you kind of realise that there is something bigger than you, you start to realise don't hurt yourself, have more respect for yourself, and you also don't harm others in a sense, not but everything falls into place. The world has meaning, has greater meaning. (C19)
6.2 Spiritual engagement	New or renewed engagement with spiritual or religious practices, helping with meaning-making and providing comfort and security	Like wow so I ran over to them and they kind of just guided me so I was just being guided by all of the spirits and stuff and they guided me up the hill into the woods. (A02)<br><br>…was actually, uh, around all of this I was sort of meditating and looking at the more spiritual aspects of my life and you know, just looking for, I was looking and seeking that help and I was, I was meditating. It, it, it brought a lot of pain but I was looking at that. (A08)<br><br>I became very um sort of what's the word, like spiritual as well. I found the church very comforting and kind of a place I would be, to trust and yeah, it just gave a sense of security for some reason. (A14)<br><br>So, you know, if it wasn't, if it wasn't for yoga, my Buddhist practice and community, who get, who don't, who, who see that there's something valuable from raw emotion, that

		there’s energy in raw emotion…My understanding of Buddhism had, had grown. Erm, I’d been introduced to it, to the practice of chanting [inaudible], to reveal your own courage and compassion and wisdom and create, create more humane connections with people. And, I thought it was amazing energy to, to nourish, ‘cos it wasn’t based on any gods or idols or who was, you know, who was, who was cool and who wasn’t. It’s universal. It’s a universal truth….the long and short of it, Buddhist practice helped [laughs]. More harmonious with myself and other people. (B09).<br><br>Every morning I put my hand on my heart and I pray and I meditate and I go into that space where it’s myself…Now I feel like now I do feel a lot more stable than I did back then and then when I go to these kinds of experiences you know, I've started to like connections have become my thing because connections are what saved me from like, connections with people, a spiritual connection and this connection between people, there's interconnection between people is essentially what saves people. (B25)<br><br>I was still a Quaker at this point so that was really helpful, so I would go each week to have silent worship and that was really good, I saw it as an opportunity to kind of pause and have my brain calm down and yeah that was really lovely. (C15)<br><br>…think also a lot, mental health is to do with emotion, for me I can only find the emotional vocabulary within church. And many things that we are struggling with are deep spiritual psychic rooms and you can't find that power and that passion in a, in anything other than a spiritual context… So for me recovery is about spirituality, it is about God, that is me and that's what works for me, it might not be the same for everyone…just pray, pray. (laughs) It works, do it, don't be scared. (C19)
