# Supplementary material for: Post-traumatic growth in mental health recovery: qualitative study of narratives
Source: BMJ Open. 2019 Jun 28;9(6):e029342. doi: 10.1136/bmjopen-2019-029342 (PMC6609070; doi:10.1136/bmjopen-2019-029342)
Supplement: Author's manuscript [file bmjopen-2019-029342.draft_revisions.pdf]

BMJ Open is committed to open peer review. As part of this commitment we make the peer review history of every article we publish publicly available.

When an article is published we post the peer reviewers' comments and the authors' responses online. We also post the versions of the paper that were used during peer review. These are the versions that the peer review comments apply to.

The versions of the paper that follow are the versions that were submitted during the peer review process. They are not the versions of record or the final published versions. They should not be cited or distributed as the published version of this manuscript.

BMJ Open is an open access journal and the full, final, typeset and author-corrected version of record of the manuscript is available on our site with no access controls, subscription charges or pay-per-view fees (http://bmjopen.bmj.com).

If you have any questions on BMJ Open's open peer review process please email info.bmjopen@bmj.com

BMJ Open

Post-traumatic growth in mental health recovery: qualitative study

Journal:	*BMJ Open*
Manuscript ID	bmjopen-2019-029342
Article Type:	Research
Date Submitted by the Author:	23-Jan-2019
Complete List of Authors:	Slade, Mike; University of Nottingham School of Health Sciences,<br>Rennick-Egglestone, Stefan; University of Nottingham School of Health Sciences, Institute of Mental Health<br>Blackie, Laura; University of Nottingham, Department of Psychology<br>Llewellyn-Beardsley, Joy; University of Nottingham School of Health Sciences, Institute of Mental Health<br>Franklin, Donna; NEON Lived Experience Advisory Panel<br>Hui, Ada; University of Nottingham School of Health Sciences<br>Thornicroft, Graham; institute of psychiatry, health service and population research<br>McGranagan, Rose; Queen Mary University of London, Unit of Social and Community Psychiatry<br>Pollock, Kristian; Nottingham University, School of Nursing<br>Priebe, Stefan; Barts and the London School of Medicine and Dentistry, University of London, Unit of Social and Community Psychiatry<br>Ramsay, Amy; Institute of Psychiatry, Psychology and Neuroscience, King's College London<br>Roe, David; University of Haifa, Department of Community Mental Health<br>Deakin, Emilia; University of Nottingham School of Health Sciences, Institute of Mental Health
Keywords:	Post-traumatic growth, MENTAL HEALTH, recovery

SCHOLARONE™
Manuscripts

Post-traumatic growth in mental health recovery: qualitative study

Mike Slade[1*]
Stefan Rennick-Egglestone[1]
Laura E. R. Blackie[2]
Joy Llewellyn-Beardsley[1]
Donna Franklin[3]
Ada Hui[1]
Graham Thornicroft[4]
Rose McGranahan[5]
Kristian Pollock[6]
Stefan Priebe[5]
Amy Ramsay[4]
David Roe[7]
Emilia Deakin[1]

[1] School of Health Sciences, Institute of Mental Health, University of Nottingham, UK
[2] School of Psychology, University of Nottingham, UK
[3] NEON Lived Experience Advisory Panel, Nottingham, UK
[4] Centre for Implementation Science, Health Service and Population Research Department, Institute of Psychiatry, Psychology and Neuroscience, King's College London, UK
[5] Unit of Social and Community Psychiatry, Queen Mary University of London
[6] School of Health Sciences, University of Nottingham, UK
[7] Department of Community Mental Health, University of Haifa, Israel

[*] Corresponding author
Email: m.slade@nottingham.ac.uk
Address: School of Health Sciences, Institute of Mental Health, University of Nottingham, Triumph Road, Nottingham NG7 2TU, UK

Word count (main text): 3,890

For peer review only - http://bmjopen.bmj.com/site/about/guidelines.xhtml

Abstract

Objectives

Post-traumatic growth, defined as positive psychological change experienced as a result of the struggle with challenging life circumstances, is under-researched in people with mental health problems. The aim of this study was to develop a conceptual framework for post-traumatic growth in the context of recovery for people with psychosis and other severe mental health problems.

Design

Qualitative cross-sectional semi-structured interviews about personal experiences of mental health recovery.

Setting

England.

Participants

Participants were adults aged over 18 and: (i) living with psychosis and not using mental health services (n=21); (ii) using mental health services and from black and minority ethnic communities (n=21); (iii) underserved, operationalised as lesbian, gay, bisexual and transgender community or complex needs or rural community (n=19); or (iv) employed in peer roles using their lived experience with others (n=16). The 77 participants comprised 42 (55%) female and 44 (57%) White British.

Results

Components of post-traumatic growth were present in 64 (83%) of recovery narratives. Six superordinate categories were identified, consistent with a view that post-traumatic growth involves learning about oneself (Self-discovery) leading to a new sense of who one is (Sense of self) and one's relationship with life (Life perspective). Observable positively-valued changes comprise a greater focus on self-management (Wellbeing)

and more importance being attached to relationships (Relationships) and spiritual or religious engagement (Spirituality). Categories are non-ordered and individuals may start from any point in this process.

Conclusions

Post-traumatic growth is often part of mental health recovery. Changes are compatible with research about growth following trauma, but with more emphasis on self-discovery, integration of illness-related experiences and active self-management of wellbeing. Trauma-related growth may be a preferable term in a mental health context. Trauma-informed mental health care could use the six identified categories as a basis for developing new approaches to supporting recovery.

Key words

Post-traumatic growth, mental health, recovery

Trial registration

Conducted as part of the NIHR NEON Study (ISRCTN11152837).

For peer review only - http://bmjopen.bmj.com/site/about/guidelines.xhtml

Article summary

Strengths and limitations of this study

- This study reports findings from a substantial qualitative investigation of post-traumatic growth in people with psychosis and other severe mental health problems
- The purposive sampling involved under-researched groups, including people with mental health problems who do not use mental health services or who are underserved by mental health services
- The involvement of eight analysts from varied backgrounds including several with lived experience of mental ill-health increases the credibility of the data analysis
- Participants were self-selecting so the views of people who do not associate with the term 'recovery' may be under-represented
- Interviewing paired family members and mental health workers would have provided new perspectives on the findings

For peer review only - http://bmjopen.bmj.com/site/about/guidelines.xhtml

Introduction

Post-traumatic growth is a relatively new area of research. The concept was introduced twenty years ago,[1] and is defined as perceptions of 'positive psychological change experienced as a result of the struggle with highly challenging life circumstances'.[2] Other terms for this phenomenon have also been used, such as benefit-finding,[3] both a coping profile and a coping outcome,[4] and positive illusions.[5] This range of terms points to the complexity of the phenomenon, with different theorists emphasising change in identity and narrative (i.e. the sense of self),[6] change in eudaimonic wellbeing (i.e. subjective quality of life),[7] and change in social/psychological resources (i.e. broadening response repertoires).[8] However, there is broad consensus over five post-traumatic growth domains: improved relations with others; identification of new possibilities for one's life; increased perception of personal strengths; spiritual growth; and enhanced appreciation of life.[2]

Post-traumatic growth is now a well-established concept in relation to 'event trauma', i.e. experience of a single traumatic event. Post-traumatic growth is widely reported in relation to event trauma and it is associated with mental health outcomes, such as depression. However, the direction of relationships between PTG and depression are not consistent across studies. For example, post-traumatic growth was found to moderate the negative association with quality of life found for both depression and post-traumatic stress in 58 Norwegian survivors interviewed two and six years after the 2004 Southeast Asia tsunami.[9] Other studies have shown that post-traumatic growth is associated with a higher level of depression, such as a two-year follow-up study of 316 survivors of the L'Aquila earthquake in Italy.[10] Similarly, higher post-traumatic growth was associated with lower post-traumatic stress disorder and higher

For peer review only - http://bmjopen.bmj.com/site/about/guidelines.xhtml

depression in 186 Iraqi students with an average of 13 war-related adversity experiences.[11] There is some evidence of a curvilinear association between depression and post-traumatic growth, for example two studies of assault survivors (n=270 in total) found survivors with low or high post-traumatic growth reported more symptoms of post-traumatic stress (both studies) and depression (one study) than those with intermediate growth levels.[12] Longitudinal research is needed to fully understand how reports of post-traumatic growth interact with depression in the recovery from event trauma.

Post-traumatic growth has relevance to health care, for example liver transplantation[13] and stroke.[14] There are several reasons why post-traumatic growth may also be relevant to psychosis and other severe mental health problems.[15] First, abuse incidence is high. In particular, childhood adversity is strongly associated with increased risk of psychosis. A meta-analysis of 18 case-control studies (n=2,048 psychosis, n=1,856 controls), 10 prospective studies (n=41,803) and 8 population-based cross-sectional studies (n=35,546) found significant associations between adversity and psychosis across all designs (OR2.78, 95%CI 5 2.34–3.31).[16] Second, comorbid depression, which is implicated in post-traumatic growth, is common. For example, around 50% of people with a diagnosis of schizophrenia also experience depression.[17] Third, both the experience of psychosis itself and the consequent experiences of societal discrimination and re-traumatisation caused by mental health system responses[18, 19] may generate trauma. Finally, some people with personal experience of psychosis report post-traumatic growth.[20, 21]

For peer review only - http://bmjopen.bmj.com/site/about/guidelines.xhtml

There is strong evidence that positive changes can be experienced after first-episode psychosis. A systematic review,[22] published initially as a scoping review,[23] identified 40 studies involving 715 participants investigating the experience of positive change after first episode psychosis. The review identified three levels of positive change. Individual-level changes included new insights and self-understanding, new meaning in life and changed values with a greater emphasis on the importance of others and less on materialism and societal expectations. At the level of others, changes included more importance attached to family and friends, more insight into who true friends were, and a greater appreciation for the value of helping others. The third level of change was spiritual, with increased levels of spirituality and religiosity, and more positive religious experiences.

There is only limited evidence about the frequency and types of post-traumatic growth in people living long-term with psychosis and other severe mental illness experiences.[24] A quantitative study of 121 people with severe mental illness using community mental health rehabilitation centres in Israel found high levels of trauma,[25] and that meaning-making and coping self-efficacy mediated post-traumatic growth experiences.[26] Three small (n=7,[27] n=7,[28] n=10[29]) qualitative studies using interpretative phenomenological analysis of semi-structured interviews all identify themes of personal growth. Current evidence indicates that growth is integral to recovery, and involves both restoration of existing aspects of identity and construction of new aspects.[30]

The extent to which (a) the five growth processes identified from event trauma research and (b) the more preliminary early psychosis-specific restorative and

For peer review only - http://bmjopen.bmj.com/site/about/guidelines.xhtml

constructive identity processes are characterising the same changes is unknown. Integration of these two sources of evidence is needed, as is investigation of the experiences of a broader range of people with long-term psychosis, including under-researched groups. The aim of this study is to develop a conceptual framework for post-traumatic growth in the context of recovery for people with psychosis and other severe mental health problems.

Methods

This research was undertaken as part of the NIHR Narrative Experiences Online (NEON) study (ISRCTN11152837, information at http://www.researchintorecovery.com/neon) between March and August 2018. Ethical Committee approval was obtained (Nottingham 2 REC 17/EM/0401). All participants provided written informed consent.

Participants

A purposive sample of under-researched populations took part. Inclusion criteria common to all groups were: aged over 18; willing to discuss experiences; able to give informed consent; and fluent in English.

Group A (Outside the system) comprised people having had self-identified experiences of psychosis and not using services. Psychosis experience prevalence estimates in the general population range from 7%[31] to 13%,[32] yet lifetime rates of psychosis, determined through contact with services, range from 0.2% (narrowly defined criteria) to 0.7% (broadly defined).[33] The experience of the many individuals who have psychosis without using services may therefore illuminate growth

processes. Additional inclusion criteria for Group A were: self-identified lifetime experience of psychosis; no use of secondary mental health services over the previous five years.

Group B (BAME) comprised people who identified as being from Black and Minority Ethnic (BAME) populations. Ethnic minority groups often have problematic relationships with services,[34] and research about recovery in these populations identifies a strong emphasis on the post-traumatic growth concepts of connectedness[35] and spirituality.[36] Additional inclusion criteria for Group B were: currently using mental health services; Black, Asian and minority ethnic community member.

Group C (Under-served) comprised people who were not well engaged with mental health services. This was operationalised for three under-served groups: people from lesbian, gay, bisexual or trans (LGBT+) communities;[37] from rural communities;[38] or with multiple complex health and social care needs.[39] Additional inclusion criteria for Group C were: experience of mental health problems in previous 10 years; no or mainly unsuccessful interactions with formal mental health services; member of LGBT+ communities OR living in an area with less than 10,000 population OR experience of at least two of homelessness, substance misuse issues or offending.

Group D: (Peer) comprised people with experience of working in statutory or voluntary roles for which lived experience is a requirement, e.g. peer support workers, trainers or researchers. Addition inclusion criteria for Group D were: working in statutory or

For peer review only - http://bmjopen.bmj.com/site/about/guidelines.xhtml

voluntary roles for which lived experience is an essential requirement; use their lived experience as a tool for engagement with other mental health service users.

Setting

Participants were recruited across England, with Groups A (Outside the system) and B (BAME) primarily from London and Groups C (Under-served) and D (Peer) primarily from the Midlands. Group A were recruited through primary care services support groups, Hearing Voices Network, and online advertising. Group B were recruited through community groups, a Recovery College and psychosis-specific secondary mental health services. Group C were recruited through community networks, voluntary sector organisations and secondary care mental health services. Group D were recruited through community groups and secondary care mental health services. Recruitment for all groups used snowball sampling.

Procedures

A preliminary coding framework (Online Supplement 1) was developed in advance of interviews, by merging existing post-traumatic growth dimensions identified in trauma populations[40] and in previous studies of post-traumatic growth in psychosis.[22, 23, 28] The preliminary coding framework was intended to establish the link between existing research and participant narratives, and comprised the code name, definition and examples drawn from the source references. An 'Other' category was added to allow the emergence of new themes.

Interviews using a narrative approach were conducted by four researchers from sociology, advocacy, public health and health psychology backgrounds. Each

For peer review only - http://bmjopen.bmj.com/site/about/guidelines.xhtml

participant took part in a 40-90 minute interview conducted in a health service or community venue. The topic guide (shown in Online Supplement 2) asked the participant to share their mental health and recovery experiences, as if it were a story, with a beginning, a middle and an end, and to include some consideration of what might happen in the future. Participants were remunerated (£20) for their time, and given options to pause or discontinue if they became distressed. Interviews were recorded, and pseudonymised transcripts were made after interviews. After the interview the researcher wrote field notes, which were included in the analysis. Interviews were conducted until theoretical saturation was achieved.

Analysis

The four coders comprised three interviewers plus one non-interviewer with an interdisciplinary background in sociology and mental health nursing. Thematic analysis was undertaken using NVivo version 11.[41] Coding was initially according to the preliminary coding framework and informed by field notes, but coders remained open to the identification of additional themes in the data. Coding involved identification and allocation of text relating to the coding framework, enabling related text to be grouped and compared, allowing identification of themes occurring within and across sources. Regular discussions between analysts explored how themes of post-traumatic growth were expressed and related to each other, allowing lower order themes to be recognised.[42] Each coder independently coded and compared the same initial transcript. Remaining transcripts were then coded separately (25% per coder).

The coding framework was then iteratively refined in meetings between the four primary coders and a wider group of four other non-interviewer analysts with expertise

For peer review only - http://bmjopen.bmj.com/site/about/guidelines.xhtml

in healthcare technologies, qualitative research, recovery research and clinical psychology. Several of the interviewers, coders and analysts also had lived experience of mental ill-health and recovery, to enhance the role of lived experience in collection and analysis of data.[43] To enhance trustworthiness, an audit trail was kept, and an interim coding framework is shown in Online Supplement 3. The conceptual framework, i.e. the final coding framework, was agreed by all coders and analysts.

Results

The sociodemographic and clinical characteristics of the 77 participants are shown in Table 1.

Insert Table 1 here

Post-traumatic growth components were coded in 64 (83%) of the 77 transcripts. The conceptual framework for post-traumatic growth in psychosis and other severe mental health problems is summarised in Table 2 with a complete version including more example coding in Online Supplement 4.

Insert Table 2 here

Major themes

Self-discovery

For peer review only - http://bmjopen.bmj.com/site/about/guidelines.xhtml

The self-discovery theme involves a fuller and deeper understanding of oneself. This involves the ability to access, accept and be mindful of difficult feelings:

> *…what was going on was an internal not an external thing.* (B04)

> *…the key to everything isn't it, accessing your emotions, not running away from them…* (C19)

The resulting self-knowledge leads to greater authenticity and being less influenced by the expectations of others

> *I feel like I know myself quite well, you know I can heal myself.* (A08)

> *When I discovered that freedom, that I didn't have to join the rat-race…that was quite liberating…* (B18)

Self-acceptance can arise from greater self-knowledge, by letting go of past difficulties and developing self-compassion

> *It's all about self-accepting, getting to know me and it helped.* (A17)

> *The key word is accepting the situation that I was in, um, and being honest with myself.* (C03)

For peer review only - http://bmjopen.bmj.com/site/about/guidelines.xhtml

Alongside these processes, participants talked about the importance of taking, or taking back, responsibility for one's own life

> *…that hit me like a, er, thunderbolt, knowing that, knowing I, that if I'm not going to help myself, no one else will help me. And that was the beginning, really, of my recovery.* (B02)

> *This was like taking a step back and looking at almost re-engineering life to take into account self-care, self-preservation and also building myself up…* (D08)

Sense of self

The development of a more positive sense of self involved integration and valuing of illness experiences. A repeated theme was pride in oneself as a person.

> *I believe in my self-worth these days…I must pat myself on the back.* (A15)

> *I think that's something I'm pretty proud of actually, that I just take people as they are.* (B21)

An important part of this pride was integrating experiences of mental ill-health so they become an accepted part of one's sense of self.

> *You don't choose the issues that you've got but you can, you can make a choice to change.* (C04)

For peer review only - http://bmjopen.bmj.com/site/about/guidelines.xhtml

I am who I am because of what happened. (D04)

Some participants moved beyond accepting to positively valuing these experiences.

What they were calling symptoms that must be eradicated, were actually part of me and so I looked behind that and said that is where, that is where my creativity comes from. (A19)

I am still me but I am a different me and I am stronger. (D05)

Life perspective

Participants identified a new or renewed appreciation of, or gratitude about, aspects of life. For some this was a general sense of appreciation.

…I'm becoming one of those ridiculously ever hopeful, ever optimistic people who say there is hope, my life is a life that is about hope... (A08)

…I am alive, I appreciate that I am alive… (C19)

For others there was a new appreciation of support received from support groups, mental health services and workers.

…it [organisation] completely changed my view of life. (A11)

For peer review only - http://bmjopen.bmj.com/site/about/guidelines.xhtml

Rehab and coming to <service name> changed my life, it's like <worker> got me on a college course, it has been absolutely wonderful… (C18)

The idea that suffering has been meaningful or worthwhile was expressed.

Wow I see where I am and I go back to then and I just think 'you didn't go through that in vain'. (A17)

…I suppose I am grateful, for want of another way of putting it, that I have lived the life I have, I have had these experiences. (D04)

New connections with political consciousness or a survivor mission to ensure others did not have similar bad experiences were identified by some participants, especially in Group B.

…I could identify with more, kind of, politicised…the personal was political and I was beginning to become aware of that on a deeper level. (B09)

And then as I came back out it was just like no, I wanted to help…I've got stuff I want to do, I want to help people... (B25)

Wellbeing

The above psychological processes were complemented by a more active engagement in managing wellbeing and lifestyle. A determination to stay well was identified.

For peer review only - http://bmjopen.bmj.com/site/about/guidelines.xhtml

…I swore from that day on, no man, money, love or beast would ever put me back into that situation again and I have stuck to it. (A15)

…I've been there, right there to the lowest of the low. And pulled myself back from it. And that's difficult to do. (C12)

This led to a greater engagement in wellbeing-related activities, including arts, music, sport, nature and learning

I think the art has given me great kind of, great kind of structure. (A01)

…I've just found learning to be so therapeutic and rewarding that I feel like I am at a point now where I can actually study and put my mind towards…doing something worthwhile. (B10)

Relationships

Many participants identified changes in relationships. For some this involved more actively choosing which relationships to continue, to re-start or to end

I got rid of this awful man in my life. (B06)

I needed to go back to a couple of my old primary school friends' houses and ask for their forgiveness for something. (B07)

For peer review only - http://bmjopen.bmj.com/site/about/guidelines.xhtml

For others, the change was in the value placed on relationships with others.

> *…it's brought the incredible closeness with people with mental health with my, with my immediate family and friends…* (A03)

> *It's been a process of learning that I needed, I need desperately, I desperately needed family, you know, people I feel safe with, to be myself.* (B09)

A greater ability to empathise with others was also identified.

> *So my purpose really is young people and even when I see the destruction that young people are going through I never blame them, I said it stems from somewhere…* (A17)

> *You have understanding, empathy…You really empathise.* (B15)

All of these relationship processes informed a desire to give back, both by supporting others in similar situations and by giving back to society. This code differs from the survivor mission and mutuality sub-themes in its emphasis on giving as intrinsically beneficial.

> *I just see myself as hopefully being a beacon to others who are, you know, struggling, and others who are finding things difficult.* (B02)

> *I want to go on and help people if I can that have been through the same thing.* (C18)

Spirituality

Participants, especially in Group A, described a deeper engagement with spirituality, religious and existential endeavours. For some, this was expressed as an enhanced spiritual awareness.

> *Now I'm sort of growing older I know they're spirit animals, I still have them and so it's the wolves that are the most powerful so I do feel protected by those.* (A18)

> *…as I went through that whole process it was like this massive opening, just kind of spiritual opening again.* (A10)

This was associated with new or renewed observable engagement in spiritual or religious practices.

> *… I was sort of meditating and looking at the more spiritual aspects of my life and…I was looking and seeking that help…* (A08)

> *…just pray, pray. (laughs) It works, do it, don't be scared.* (C19)

Discussion

Post-traumatic growth concepts were identified in 64 (83%) of the interviews with 77 diverse participants describing their mental health recovery. The six superordinate categories are not ordered, but one narrative consistent with the results is that post-traumatic growth involves learning about oneself (Self-discovery) leading to a new sense of who one is (Sense of self) and one's relationship with life (Life perspective). Observable positively-valued changes are a greater focus on self-management (Wellbeing) and more importance being attached to relationships (Relationships) and spiritual / religious engagement (Spirituality). Individuals may start from any point in this narrative, so an alternative description would be that individuals experience a change in their Life perspective, enabling an alternative Sense of self to be developed, which facilitates new kinds of Self-Discovery. The conceptual framework is compatible with the five growth processes identified in event trauma research, but participants also experienced changes in (a) self-discovery, (b) sense of self, specifically including integration of illness-related experiences into identity and (c) the importance they attached to active self-management of well-being. Many participants were currently struggling with adversity so the term 'post-traumatic' may need amending, perhaps to 'trauma-related'.

The strengths of the study include the relatively large sample size for a qualitative study, the purposive sampling of diverse and under-researched participant groups, and the large analyst team (n=8) bringing multiple perspectives to enhance trustworthiness of data analysis. Weaknesses include the limited geographical spread mainly from two parts of England, an assumption that trauma was present and therefore not coded for by analysts, and the absence of wider perspectives such as paired family or mental health staff interviews. Identifying a change as positive was

For peer review only - http://bmjopen.bmj.com/site/about/guidelines.xhtml

not always straightforward, so change experiences which in this study were not coded as positive psychological change may merit further investigation. Examples which were not coded as post traumatic growth include alternative methods of getting well, escapism, knowing where to access support, cultural differences and environmental factors. Recovering from ‘trauma’ was not the frame of reference used by many participants in telling their stories, so this represents primarily a researcher framing of experiences. Relatedly, the extent to which participants attributed changes to post-traumatic growth, and the extent to which their formulations were derived from cultural tropes, were not explored. An alternative analysis approach would have involved inductive coding followed by comparison with existing research. Finally, the cross-sectional coding inevitably abstracts from complex and contextualised stories, so may not capture the ambivalent and conflicting perspectives common in narratives.

This study provides further evidence that post-traumatic growth concepts are relevant to many people living with psychosis and other severe mental health problems. The concept of recovery in mental health has come to mean living as well as possible, whether or not symptoms are present. Recovery thus differs from the traditional clinical priorities of symptom remission and functional restoration,[44] and a systematic review identified five recovery processes: Connectedness, Hope, Identity, Meaning and Empowerment (CHIME Framework).[35] A mental health service orientation towards supporting this understanding of recovery is recommended internationally,[45, 46] and central to national policy in many countries.[47-50] A recovery orientation involves system transformation[51] requiring new clinical approaches including a greater emphasis on supporting strengths,[52-54] self-management,[55, 56] hope,[57, 58] and wellbeing,[59-61] more use of new interventions such as positive psychology,[62, 63] Recovery Colleges[64-67] and

For peer review only - http://bmjopen.bmj.com/site/about/guidelines.xhtml

peer support,[68, 69] and a greater focus on human rights.[70, 71] The current study supports the case that trauma-informed approaches to mental health care should be added to this list of recovery-supporting innovations.[72]

The conceptual framework provides a theoretical foundation for work to support post-traumatic growth in mental health. Encouraging post-traumatic growth is an important contribution to supporting recovery. For example, a study of post-traumatic growth and recovery in 34 people experiencing first episode psychosis concluded that '*people with early psychosis may benefit from disclosing their experiences of psychosis, including those aspects that were traumatic, as this may support the processes of recovery and post-traumatic growth*' (p.213).[73] A clinical implication is that it is important to reinforce efforts by service users to find their own personally satisfactory meaning of their experiences, rather than simply encouraging the adoption of a clinical explanatory model. Similarly, supporting service users to engage in wellbeing self-management, to actively choose relationships and to explore spiritual development should be a significant clinical focus.

The relationship between mental health recovery and post-traumatic growth is unclear, as is the extent to which they are the same or overlapping but distinct phenomena. Prospective longitudinal research is needed to investigate if there is a temporal relationship between positive psychological change and development of an identity as a 'person in recovery' for different clinical sub-populations, and how these change in the context of crisis or relapse. The implications for mental health care processes also need to be investigated, specifically identifying clinical sub-populations for whom a post-traumatic growth approach is particularly indicated, and developing and

For peer review only - http://bmjopen.bmj.com/site/about/guidelines.xhtml

evaluating manualised treatment approaches which support the identified positive psychological changes. What is clear is that moving forward from severe mental health problems is not simply a question of taking treatment as prescribed, but a far more active process of engaging and re-engaging in the search for meaning for oneself, learning to manage the demands of living well, and finding one's place in the world.

For peer review only - http://bmjopen.bmj.com/site/about/guidelines.xhtml

References

1. Tedeschi R. Violence transformed: posttraumatic growth in survivors and their societies. *Aggressive and Violent Behavior* 1999; **4**: 319-41.
2. Tedeschi R, Calhoun L. Posttraumatic growth: Conceptual foundations and empirical evidence. *Psychological Inquiry* 2004; **15**: 1–18.
3. Tomich P, Helgeson V. Is finding something good in the bad always good? Benefit finding among women with breast cancer. *Health Psychol* 2004; **23**: 16–23.
4. Zoellner T, Maercker A. Posttraumatic growth in clinical psychology - a critical review and introduction of a two component model. *Clin Psychol Rev* 2006; **26**: 626-53.
5. Taylor S, Armor D. Positive illusions and coping with trauma. *J Pers* 1996; **64**: 873–98.
6. Pals J, McAdams D. The transformed self: A narrative understanding of posttraumatic growth. *Psychological Inquiry* 2004; **15**: 65–9.
7. Joseph S, Linley P. Positive adjustment to threatening events: An organismic valuing theory of growth through trauma. *Review of General Psychology* 2005; **9**: 262–80.
8. Hobfoll S, Hall B, Canetti-Nisim D, Galea S, Johnson R, Palmieri P. Refining our understanding of traumatic growth in the face of terrorism: Moving from meaning cognitions to doing what is meaningful. *Applied Psychology: An International Review* 2007; **56**: 345–66.
9. Siqveland J, Nygaard E, Hussain A, Tedeschi R, Heir T. Posttraumatic growth, depression and posttraumatic stress in relation to quality of life in tsunami survivors: a longitudinal study. *Health and Quality of Life Outcomes* 2015; **13**: 18.

For peer review only - http://bmjopen.bmj.com/site/about/guidelines.xhtml

10. Bianchini V, Giusti L, Salza A, et al. Moderate Depression Promotes Posttraumatic Growth (Ptg): A Young Population Survey 2 Years after the 2009 L'Aquila Earthquake. *Clinical Practice & Epidemiology in Mental Health* 2017; **13**: 10-9.
11. Magruder M, Kılıç C, Koryürek M. Relationship of posttraumatic growth to symptoms of posttraumatic stress disorder and depression: A pilot study of Iraqi students. *International Journal of Psychology* 2015; **50**: 402-6.
12. Kleim B, Ehlers A. Evidence for a Curvilinear Relationship Between Posttraumatic Growth and Posttrauma Depression and PTSD in Assault Survivors. *J Trauma Stress* 2009; **22**: 45-52.
13. Pérez-San-Gregorio M, Martín-Rodríguez A, Borda-Mas M, et al. Post-traumatic growth and its relationship to quality of life up to 9 years after liver transplantation: a cross-sectional study in Spain. *BMJ Open* 2017; **7**: e017455.
14. Shipley J, Luker J, Thijs V, Bernhardt J. The personal and social experiences of community-dwelling younger adults after stroke in Australia: a qualitative interview study. *BMJ Open* 2018; **8**: e023525.
15. Dunkley J, Bates G, Foulds M, Fitzgerald P. Understanding Adaptation to First-Episode Psychosis: The Relevance of Trauma and Posttraumatic Growth. *Australasian Journal of Disaster and Trauma Studies* 2007; **2007-1**: 1-16.
16. Varese F, Smeets F, Drukker M, et al. Childhood Adversities Increase the Risk of Psychosis: A Meta-analysis of Patient-Control, Prospective- and Cross-sectional Cohort Studies. *Schizophr Bull* 2012; **38**(4): 661-71.
17. Buckley P, Miller, B., Lehrer, D., Castle, D. Psychiatric Comorbidities and Schizophrenia. *Schizophr Bull* 2009; **35**: 383-402.
18. Bloom S, Farragher B. Destroying Sanctuary: The Crisis in Human Service Delivery Systems. Oxford: Oxford University Press; 2010.

For peer review only - http://bmjopen.bmj.com/site/about/guidelines.xhtml

19. Priebe S, Bröker M, Gunkel S. Involuntary Admission and Posttraumatic Stress Disorder Symptoms in Schizophrenia Patients. *Compr Psychiatry* 1998; **39**: 220-4.

20. Chadwick PK. Schizophrenia: The Positive Perspective. London: Routledge; 1997.

21. Roe D, Chopra M. Beyond Coping with Mental Illness: Towards Personal Growth. *Am J Orthopsychiatry* 2003; **73**: 334-44.

22. Jordan G, MacDonald K, Pope M, Schorr E, Malla A, Iyer S. Positive Changes Experienced After a First Episode of Psychosis: A Systematic Review. *Psychiatr Serv* 2018; **69**: 84-99.

23. Jordan G, Pope, M., Lambrou, A., Malla, A., Iyer, S. Post-traumatic growth following a first episode of psychosis: a scoping review. *Early Intervention in Psychiatry* 2017; **11**: 187-99.

24. Slade M, Blackie L, Longden E. Personal growth in psychosis. *World Psychiatry* 2019; **18**: 29-30.

25. Mazor Y, Gelkopf, M., Mueser, K., Roe, D. Posttraumatic growth in psychosis. *Frontiers in Psychiatry* 2016; **7**: 202.

26. Mazor Y, Gelkopf M, Roe D. Posttraumatic growth among people with serious mental illness, psychosis and posttraumatic stress symptoms. *Compr Psychiatry* 2018; **81**: 1-9.

27. Dixon L, Sanderson C, Holt L. A Weird but Interesting Journey: Personal Traumatic Growth for Individuals with Hallucinations. *Journal of Psychology and Psychotherapy* 2018; **8**: 343.

For peer review only - http://bmjopen.bmj.com/site/about/guidelines.xhtml

28. Mapplebeck C, Joseph S, Sabin-Farrell R. An Interpretative Phenomenological Analysis of Posttraumatic Growth in People With Psychosis. *Journal of Loss and Trauma* 2015; **20**: 34-45.

29. Attard A, Larkin M, Boden Z, Jackson C. Understanding Adaptation to First Episode Psychosis Through the Creation of Images. *Journal of Psychosocial Rehabilitation and Mental Health* 2017; **4**: 73-88.

30. Dunkley J, Bates G. Recovery and adaptation after first-episode psychosis: The relevance of posttraumatic growth. *Psychosis* 2015; **7**: 130-40.

31. Linscott R, van Os J. An updated and conservative systematic review and meta-analysis of epidemiological evidence on psychotic experiences in children and adults: on the pathway from proneness to persistence to dimensional expression across mental disorders. *Psychol Med* 2013; **43**: 1133-49.

32. Beavan V, Read, J., Cartwright, C. The prevalence of voice-hearers in the general population: A literature review. *Journal of Mental Health* 2011; **20**: 281-92.

33. Kendler KS, Gallagher TJ, Abelson JM, Kessler RC. Lifetime prevalence, demographic risk factors, and diagnostic validity of nonaffective psychosis as assessed in a US community sample. The National Comorbidity Survey. *Arch Gen Psychiatry* 1996; **53**: 1022-31.

34. Ghali S, Fisher HL, Joyce J, et al. Ethnic variations in pathways into early intervention services for psychosis. *Br J Psychiatry* 2013; **202**(4): 277-83.

35. Leamy M, Bird V, Le Boutillier C, Williams J, Slade M. A conceptual framework for personal recovery in mental health: systematic review and narrative synthesis. *Br J Psychiatry* 2011; **199**: 445-52.

For peer review only - http://bmjopen.bmj.com/site/about/guidelines.xhtml

36. Whitley R. Ethno-Racial Variation in Recovery From Severe Mental Illness: A Qualitative Comparison. *Canadian Journal of Psychiatry* 2016; **61**: 340-7puschner.

37. Hudson-Sharp N, Metcalf H. Inequality among lesbian, gay bisexual and transgender groups in the UK: a review of evidence. London: NIESR, 2016.

38. Gunnell D, Wheeler B, Chang S, Thomas B, Sterne J, Dorling D. Changes in the geography of suicide in young men: England and Wales 1981–2005. *J Epidemiol Community Health* 2012; **66**: 536-43.

39. Kuluski K, Ho J, Hans K, Nelson M. Community Care for People with Complex Care Needs: Bridging the Gap between Health and Social Care. *International Journal of Integrated Care* 2017; **17**: 1-11.

40. Jayawickreme E, Blackie, L. Post-traumatic growth as positive personality change: Evidence, controversies and future directions. *European Journal of Personality* 2014; **28**: 312-31.

41. QSR International Pty Ltd. NVivo qualitative data analysis Software Version 11.0. 2015.

42. Bazeley P. Qualitative Data Analysis, Practical Strategies. London: Sage; 2013.

43. Jennings H, Slade M, Bates P, Munday E, Toney R. Best practice framework for Patient and Public Involvement (PPI) in collaborative data analysis of qualitative mental health research: methodology development and refinement. *BMC Psychiatry* 2018; **18**: 213.

44. Palmquist L, Patterson S, O'Donovan A, Bradley G. Protocol: A grounded theory of ‘recovery’—perspectives of adolescent users of mental health services. *BMJ Open* 2017; **7**: e015161.

For peer review only - http://bmjopen.bmj.com/site/about/guidelines.xhtml

45. World Health Organization. Mental Health Action Plan 2013-2020. Geneva: WHO; 2013.

46. Slade M, Amering M, Oades L. Recovery: an international perspective. *Epidemiol Psichiatr Soc* 2008; **17**(2): 128-37.

47. HM Government. No health without mental health. Delivering better mental health outcomes for people of all ages. London: Department of Health; 2011.

48. Mental Health Commission of Canada. Changing directions, changing lives: The mental health strategy for Canada. Calgary: Mental Health Commission of Canada; 2012.

49. Department of Health. The Fifth National Mental Health and Suicide Prevention Plan. Canberra: Commonwealth of Australia; 2017.

50. Mental Health Commission. Strategic Plan 2016 - 2018. Dublin: Mental Health Commission; 2017.

51. Slade M, Amering M, Farkas M, et al. Uses and abuses of recovery: implementing recovery-oriented practices in mental health systems. *World Psychiatry* 2014; **13**: 12-20.

52. Tse S, Tsoi, E., Hamilton, B., O'Hagan, M., Shepherd, G., Slade, M., Whitley, R., Petrakis, M. Uses of Strength-Based Interventions for people with serious mental illness: A critical review. *Int J Soc Psychiatry* 2016; **62**: 281-91.

53. Priebe S, Omer S, Giacco D, Slade M. Resource-oriented therapeutic models in psychiatry – A conceptual review. *Br J Psychiatry* 2014; **204**: 256-61.

54. Bird V, Le Boutillier C, Leamy M, et al. Assessing the strengths of mental health service users - systematic review. *Psychological Assessment* 2012; **24**: 1024-33.

For peer review only - http://bmjopen.bmj.com/site/about/guidelines.xhtml

55. Cook JA, Copeland ME, Jonikas JA, et al. Results of a randomized controlled trial of mental illness self-management using Wellness Recovery Action Planning. *Schizophr Bull* 2012; **38**(4): 881-91.

56. Slade M. Implementing shared decision making in routine mental health care. *World Psychiatry* 2017; **16**: 146-53.

57. Kirst M, Zerger, S., Harris, D., Plenert, E., Stergiopoulos, V. The promise of recovery: narratives of hope among homeless individuals with mental illness participating in a Housing First randomised controlled trial in Toronto, Canada. *BMJ Open* 2014; **4**: e004379.

58. Schrank B, Bird V, Rudnick A, Slade M. Determinants, self-management strategies and interventions for hope in people with mental disorders: systematic search and narrative review. *Soc Sci Med* 2012; **74**: 554-64.

59. Slade M, Oades L, Jarden A, editors. Wellbeing, Recovery and Mental Health. Cambridge: Cambridge University Press; 2017.

60. Schrank B, Riches S, Bird V, Murray J, Tylee A, Slade M. A conceptual framework for improving well-being in people with a diagnosis of psychosis. *Epidemiology and Psychiatric Sciences* 2014; **23**: 377-87.

61. Schrank B, Riches S, Coggins T, Rashid T, Tylee A, Slade M. WELLFOCUS PPT – modified Positive Psychotherapy to improve well-being in psychosis: study protocol for pilot randomised controlled trial. *Trials* 2014; **15**: 202.

62. Slade M, Brownell T, Rashid T, Schrank B. Positive Psychotherapy for Psychosis. Hove: Routledge; 2017.

63. Slade M. Mental illness and well-being: the central importance of positive psychology and recovery approaches. *BMC Health Services Research* 2010; **10**: 26.

For peer review only - http://bmjopen.bmj.com/site/about/guidelines.xhtml

64. Whitley R, Shepherd G, Slade M. Recovery Colleges as a mental health innovation. *World Psychiatry* in press.
65. Toney R, Knight J, Hamill K, et al. Development and evaluation of a Recovery College fidelity measure. *Canadian Journal of Psychiatry*: DOI:10.1177/0706743718815893.
66. Crowther A, Taylor A, Toney R, et al. The impact of Recovery Colleges on mental health staff, services and society. *Epidemiology and Psychiatric Sciences* in press.
67. Toney R, Elton D, Munday E, et al. Mechanisms of action and outcomes for students in Recovery Colleges. *Psychiatr Serv* 2018; **69**: 1222-9.
68. Puschner B. Peer support and global mental health. *Epidemiology and Psychiatric Sciences* 2018; **27**: 413-4.
69. Pitt V, Lowe D, Hill S, et al. Consumer-providers of care for adult clients of statutory mental health services. *Cochrane Database of Systematic Reviews* 2013; (3).
70. Funk M, Drew N. WHO QualityRights: transforming mental health services. *Lancet Psychiatry* 2017; **4**: 826-7.
71. United Nations General Assembly. Report of the Special Rapporteur on the right of everyone to the enjoyment of the highest attainable standard of physical and mental health. New York: Human Rights Council; 2017.
72. Sweeney A, Clement S, Filson B, Kennedy A. Trauma-informed mental healthcare in the UK: what is it and how can we further its development? *Mental Health Review Journal* 2016; **21**: 174-92.
73. Pietruch M, Jobson, L. Posttraumatic growth and recovery in people with first episode psychosis: an investigation into the role of self-disclosure. *Psychosis* 2012; **4**: 213-23.

For peer review only - http://bmjopen.bmj.com/site/about/guidelines.xhtml

For peer review only - http://bmjopen.bmj.com/site/about/guidelines.xhtml

Patient and Public Involvement statement

Patients were involved in acquisition of funding, both through a Lived Experience Advisory Panel informing the design and through involvement as applicants. Several interviewers and analysts had their own lived experience of mental ill-health and recovery, in addition to their professional training. The NEON Lived Experience Advisory Panel (LEAP) comprising ten members with lived experience of mental ill-health and recovery informed the ethics application, trained the interviewers, informed the topic guide and supported access to Group D. A LEAP member was involved in interpretation of the findings and as a co-author of this paper. The LEAP are leading the writing of a guide to sharing stories, which will be informed by this research.

Funding statement

This article is independent research funded by the National Institute for Health Research (NIHR) under its Programme Grants for Applied Research Programme (Narrative Experiences ONline (NEON) Programme, RP-PG-0615-20016). MS acknowledges the financial support of the NIHR Nottingham Biomedical Research Centre (BRC). GT is supported by NIHR Collaboration for Leadership in Applied Health Research and Care South London (King's College London NHS Foundation Trust), Department of Health via the NIHR BRC and Dementia Unit (South London and Maudsley NHS Foundation Trust in partnership with King's College London and King's College Hospital NHS Foundation Trust), European Union Seventh Framework Programme (FP7/2007-2013, Emerald project), National Institute of Mental Health (National Institutes of Health R01MH100470, Cobalt study) and UK Medical Research Council (MR/S001255/1 Emilia; MR/R023697/1 Indigo Partnership) awards. The views expressed are those of the authors and not necessarily those of the NHS, the NIHR, the Department of Health and Social Care or other supporting funders.

Competing interests statement

None declared

Author statement

MS and LB made a substantial contribution to the conception or design of the work. All authors contributed to the acquisition, analysis, or interpretation of data for the work. All authors were involved in drafting the work or revising it critically for important intellectual content, and gave final approval of the version to be published. All authors agree to be jointly accountable for all aspects of the work.

Data sharing statement

Data available from corresponding author.

Licensing statement

I, the Submitting Author has the right to grant and does grant on behalf of all authors of the Work (as defined in the below author licence), an exclusive licence and/or a non-exclusive licence for contributions from authors who are: i) UK Crown employees; ii) where BMJ has agreed a CC-BY licence shall apply, and/or iii) in accordance with the terms applicable for US Federal Government officers or employees acting as part of their official duties; on a worldwide, perpetual, irrevocable, royalty-free basis to BMJ Publishing Group Ltd ("BMJ") its licensees and where the relevant Journal is co-owned by BMJ to the co-owners of the Journal, to publish the Work in BMJ Open and any other BMJ products and to exploit all rights, as set out in our licence.

For peer review only - http://bmjopen.bmj.com/site/about/guidelines.xhtml

The Submitting Author accepts and understands that any supply made under these terms is made by BMJ to the Submitting Author unless you are acting as an employee on behalf of your employer or a postgraduate student of an affiliated institution which is paying any applicable article publishing charge ("APC") for Open Access articles. Where the Submitting Author wishes to make the Work available on an Open Access basis (and intends to pay the relevant APC), the terms of reuse of such Open Access shall be governed by a Creative Commons licence – details of these licences and which Creative Commons licence will apply to this Work are set out in our licence referred to above.

For peer review only - http://bmjopen.bmj.com/site/about/guidelines.xhtml

Table 1: Clinical and sociodemographic characteristics of participants (n=77)

Characteristic	Total	Group A (Outside the system)	Group B (BAME)	Group C (Under-served)	Group D (Peer)
n (%)	*77 (100)*	*21 (27)*	*21 (27)*	*19 (25)*	*16 (21)*
**Gender** n (%)					
Female	42 (55)	14 (67)	11 (53)	8 (42)	9 (56)
Male	30 (39)	6 (29)	9 (43)	9 (47)	6 (38)
Other / prefer not to say	5 (6)	1 (5)	1 (5)	2 (11)	1 (6)
**Ethnicity** n (%)					
White British	44 (57)	12 (57)	0 (0)	18 (95)	14 (88)
Black British	5 (6)	2 (10)	3 (14)	0 (0)	0 (0)
Black African / Caribbean	4 (5)	1 (5)	3 (14)	0 (0)	0 (0)
White Other	5 (6)	2 (10)	1 (5)	0 (0)	2 (13)
White and Black African / Caribbean	4 (5)	0 (0)	4 (19)	0 (0)	0 (0)
Asian / Mixed white Asian	4 (5)	0 (0)	4 (19)	0 (0)	0 (0)
Other	5 (6)	2 (10)	3 (14)	0 (0)	0 (0)
Prefer not to say	6 (8)	2 (10)	3 (14)	1 (5)	0 (0)
**Age** (years)					
18-25	4 (5)	0 (0)	0 (0)	3 (16)	1 (6)
25-34	16 (21)	3 (14)	6 (29)	4 (21)	3 (19)
35-44	16 (21)	5 (24)	4 (19)	4 (21)	3 (19)
45-54	30 (39)	8 (38)	9 (43)	6 (32)	7 (43)
55+	5 (6)	4 (19)	0 (0)	0 (0)	1 (6)
Prefer not to say	6 (8)	1 (5)	2 (10)	2 (11)	1 (6)
**Sexual orientation**					
Heterosexual	49 (64)	15 (71)	14 (67)	6 (32)	14 (88)
LGBT+	18 (23)	3 (14)	4 (19)	9 (47)	2 (13)
Prefer not to say	10 (13)	3 (14)	3 (14)	4 (21)	0 (0)
**Primary diagnosis**					
Schizophrenia or other psychosis	11 (14)	5 (24)	4 (19)	2 (11)	0 (0)

For peer review only - http://bmjopen.bmj.com/site/about/guidelines.xhtml

Bipolar disorder / cyclothymia	16 (21)	8 (38)	1 (5)	3 (16)	4 (25)
Mood disorder, e.g. anxiety, depression, dysthymia	15 (19)	1 (5)	4 (19)	4 (21)	6 (38)
Other, e.g. ADHD, personality disorder, substance abuse, autism	7 (9)	0 (0)	2 (10)	3 (16)	2 (13)
Prefer not to say	28 (36)	7 (33)	10 (48)	7 (37)	4 (25)

Table 2: Final conceptual framework for post-traumatic growth in psychosis and other severe mental health conditions

Type of growth	Definition of the positively perceived change
**1. Self-discovery**	**Having a fuller and deeper understanding of oneself**
1.1 Emotional life	Discovering or re-discovering how to access, accept and be mindful of inner emotional life and difficult feelings
1.2 Self-knowledge	Knowing oneself better, being more authentic and not being as shaped by the expectations of others
1.3 Self-acceptance	Grieving and letting go of the past, and developing self-compassion
1.4 Self-responsibility	Taking (back) responsibility for one's own life
**2. Sense of self**	Development of a more positive sense of self, including integration and valuing of illness experiences
2.1 Pride in self	Taking pride in oneself, including personal strengths and achievements
2.2 Integration of experiences	Illness experiences become an accepted part of one's sense of self
2.3 Valuing of experiences	Finding positives in the experience of illness
**3. Life perspective**	**New or renewed appreciation of or gratitude about aspects of life**
3.1 Appreciation of life	Appreciation for life and the importance of hopefulness
3.2 Appreciation of support	Gratitude for support received from services
3.3 Meaningful suffering	Gratitude that suffering was meaningful and not in vain
3.4 Survivor mission	New growth of political consciousness or use of illness experiences to benefit others
**4. Wellbeing**	**More active engagement in, and management of, one's own wellbeing and lifestyle**
4.1 Motivation	Increased determined to stay well, self-manage and not return to a bad situation
4.2 Being active	More engagement in the arts, music, sport, nature and learning
**5. Relationships**	**More actively choosing and valuing relationships with others**
5.1 Choosing relationships	Actively choosing relationships to continue, to re-start or to end
5.2 Valuing relationships	Placing more value on relationships with others
5.3 Empathy	Enhanced ability to empathise with others
**6 Spirituality**	**Deeper engagement with spirituality, religious and existential endeavours**
6.1 Spiritual awareness	Increased awareness of the presence of something greater than oneself making a positive contribution by providing meaning
6.2 Spiritual engagement	New or renewed engagement with spiritual or religious practices, helping with meaning-making and providing comfort and security

For peer review only - http://bmjopen.bmj.com/site/about/guidelines.xhtml

Online Supplement 1

Preliminary coding framework for post-traumatic growth in psychosis and other severe mental health conditions

#	Code	Definition	Examples
1	Strengths	New or improved use of personal strengths or resources	More resilience, confidence, self-efficacy, skills at navigating life challenges, inner strength, determination
2	Relationships	Improved relationships with others	Better connection with friends or family, new meaningful relationships
3	Purpose	New possibilities and purpose in life	Fresh directions, e.g. career, leisure pursuits; finding a place or role in society
4	Life appreciation	Greater or altered appreciation of life	Finding new meaning in experiences, seizing opportunities, finding new life purpose or direction
5	Spirituality	More engagement with spirituality or existential questions	New spiritual practice, (re-)connecting with an organised religion, deeper life meaning
6	Character	Developing positive character traits	More creativity, compassion for others
7	Lifestyle	Making positive lifestyle changes	Better sleep, more activity, healthier relationship with food / drink, developing new interests, choosing healthier relationships
8	Identity integration	Integration of experience of psychosis into identity	Psychosis experiences as shaping who the person is now, psychosis being part but not all of identity, more insight or clarity
9	Self-acceptance	More self-acceptance and awareness	More self-compassion, positive sense of self
10	Other	Other forms of post-traumatic growth not coded above	

For peer review only

Online Supplement 2

NB The interview was in two parts. In Part 1 the participant was asked to tell their story of recovery. In Part 2, follow-up questions were asked about the impact of their stories on others. The topic guide for Part 1 did not change, and only data from Part 1 (i.e. the participant's open narrative) are reported in the current study.

Interview topic guide

START OF TOPIC GUIDE

Preparation

Before the participant arrives, allocate a unique identifier (UID) to the participant, and write it into a blank Informed Consent Form.

Introduction

- Introduce the interviewer facilitator
- Ensure the participant has read the information sheet and understands that participation is voluntary and that they are free to withdraw at any time
- Explain the aims and purpose of the activity and give a brief description of the interview structure. Tell the participant they can decide whether their story is used in the later part of the NEON study, emphasising that this may mean other people beyond the study team may see it.
- Provide an opportunity for participant to ask any questions, and then obtain written consent through the Informed Consent Form
- Describe digital recording of the interview. Clarify whether the participant wants to be video-recorded or audio-recorded.
- Turn on the recorder. Read out the UID so that it is recorded.

Questions

Part 1

Ask the participant to describe their own story of recovery. Do not use language (e.g. 'psychosis') which might be leading – refer to e.g. 'mental health difficulties'. Give them plenty of time, listen carefully. Reply if asked (e.g. "Is this okay?" – "Yes, you're doing great") and use minimal prompts if needed (e.g. "Do go on") but try to let the participant tell their story in their own words.

Part 2

Follow up with questions about the narrative, e.g.

1. Who have you shared your story with, and why?
2. What was the impact on the recipient and on you? If there was a particularly powerful part of their narrative, ask specifically about the impact of that part of their story.
4. Do you sometimes hold back some aspects of your story? If so, how do you decide what and when to hold back?

For peer review only

3. Has anyone else shared a recovery narrative with you? What parts of someone else's story made an impact? Why do you think that part made an impact?
4. How has the way you have told your story changed over time? Why do you think this is?

If the participant becomes distressed during the interview, ask if they would like to take a break or stop. If the participant reveals information which is of concern and may need reporting i.e. potential risks to another person or to themselves, or criminal behaviour, then continue the interview if you feel comfortable to, but discuss these with the PI at the earliest opportunity and where appropriate report accordingly.

End of interview

Explain the interview is now over and ask if the participant has any final questions. Give information about timeline for the study and how publications can be accessed. Thank the participant for their participation.

END OF TOPIC GUIDE

For peer review only

Online Supplement 3

Interim coding framework for post-traumatic growth in psychosis and other severe mental health conditions

#	Code	Definition	Examples
1	Strengths	New or improved recognition or use of personal strengths or resources	More resilience, confidence, self-efficacy, skills at navigating life challenges, inner strength, determination, positive character traits
2	Relationships	Improved relationships with others	Better connection with friends or family, new meaningful relationships, moving away from unhealthy relationships
3	Purposefulness	New possibilities and purpose in life	Fresh directions, e.g. career, leisure pursuits; finding a place or role in society, social or political activism.
4	Life appreciation	Greater or altered appreciation of or reflection on life	Fresh perspectives, new meaning-making framework. Finding new meaning in experiences, seizing opportunities, finding new life purpose or direction, new appreciation for informal or formal learning, new or different education or career development
5	Spirituality	More engagement with spirituality or existential questions	New spiritual practice, (re-)connecting with an organised religion, deeper life meaning
6	Self-discovery	Having a changed, fuller and deeper understanding of oneself and how to live in the world	More creativity, compassion for others, increased empathy for others, new skills
7	Lifestyle	Making positive lifestyle changes to maintain wellbeing and support self-care	Better sleep, more activity, healthier relationship with food / drink, developing new interests, choosing healthier relationships, changing social environment
8	Identity integration	Integration and sense-making of experience of mental health issues into identity	Mental health experiences as shaping who the person is now, psychosis or other mental health experiences being part but not all of identity, more insight or clarity, re-framing experiences, choosing other explanatory frameworks to make sense of experiences
9	Self-acceptance	More self-acceptance and self-compassion	More self-compassion, positive sense of self, less internalised stigma
10	Other	Other forms of post-traumatic growth not coded above	

For peer review only

Online Supplement 4

Final conceptual framework for post-traumatic growth in psychosis and other severe mental health conditions

**Type of growth**	**Definition of the positively perceived change**	**Illustrative quotes**
**1. Self-discovery**	**Having a fuller and deeper understanding of oneself**	
1.1 Emotional life	Discovering or re-discovering how to access, accept and be mindful of inner emotional life and difficult feelings	But what I will say is that, er, I found that the, my, what was going on was an internal not an external thing. Because before that I, I've moved about nine times, I'd move, I'd just move somewhere else and thought it's going to be different now. At one point I moved to the States, you know? And I'd be in the same situation in the States that I'd be in England. (B04)<br><br>There was just a point where I was just like I forgot how to feel, I forgot how to be hungry, I forgot how to feel tired, I forgot how to be exhausted, I forgot how to feel fear and then I realised oh my fuck I need to feel fear, you know, I need these things, these things are really fucking important, you know that a human being needs fear, we need tiredness, we need hunger…I moved to Hong Kong and it found me again and I realised it wasn't really my situation, it was me, it was something inside me that was drawing this energy towards me and there I, unless I changed the noise inside, the noise outside is never going to go away. (B25)<br><br>And I think that is the key to everything isn't it, accessing your emotions, not running away from them, which a lot of people in mental suffering with mental health are trying to do because it is overwhelming and painful. So it is about yeah feeling and not feeling somebody else's or not, following somebody else and not being scared to be who you are and be different I think. (C19)

For peer review only

1.2 Self-knowledge	Knowing oneself better, being more authentic and not being as shaped by the expectations of others	I feel like I know myself quite well, you know I can heal myself. And uh, these are things that people actually take for granted but took me a very long time to connect to how are you feeling you know and what do you want right now? (A08)<br><br>I think you find out who you are and sort of how you have to live to keep yourself sane in a way. (A14)<br><br>When I discovered that freedom, that I didn't have to join the rat-race of having a car, wife and three kids, you know, having a job, that was quite liberating… I was constrained by what people wanted me to do, instead of what I wanted to do. (B18)<br><br>People or old friends that would say…you've got to stop being a bit like this, actually I would take that and think to myself am I? Am I a bit like that? And question what they've said and then if I thought well no actually I'm not like that anymore, you know, then I've turned around and said well actually, you know, that's wrong, you know, because that's, that's, that's not me and you can't tell me I am something if that's not what I am. You know, well you can but you know I'm not gonna listen to you. And uh you know by, by questioning uh what people was saying, uh sort of, uh, removed that vulnerability I think, and um, by removing vulnerability has allowed me to feel more in control of myself and um, who I am. (C03)
1.3 Self-acceptance	Grieving and letting go of the past, and developing self-compassion	I grieve all the hopes that are not there, you know things that haven't worked out, and I try to accept what is there and say well this is it, this is the thing that is real, maybe the other things were a little fantastical… And so I still talk to myself a lot but it's not now, it's, it's, it's me comforting myself and actually communicating with me, you know, just sort of, um it's that thing of having learned to love myself. (A08)<br><br>I started going to loads of classical music concerts which I'd really shunned because I felt like I'd really failed as I'm not playing the violin anymore, I should be, I've really failed, I'm not good as the other people who have made it their profession. Suddenly I was like, God that doesn't matter, that is just so irrelevant, like classical music and music generally is so beautiful I just want to go and listen to it. (A10)

		It's all about self-accepting, getting to know me and it helped. (A17)<br><br>I feel like I'm at peace with a lot of the, erm, depression that I felt over things that have happened in the past. (B01)<br><br>The key word is accepting the situation that I was in, um, and being honest with myself. (C03)
1.4 Self-responsibility	Taking (back) responsibility for one's own life	Yeah, because in my own mindset and recovery…I had to become interested in the problem because prior to that my outlook on recovery in mental health was based on seeing a Consultant Psychiatrist and they will have the answer because that is the predominant feeling, patient/doctor relationship, you see a doctor, you trust what they say and then I had to become interested after I realised that the doctors can't fix me. So when I became interested I became open to learning. (A21)<br><br>The one thing he did when he came, when he saw, he said, 'I'm not going to help you do one thing in this flat unless you start doing it for yourself. If you make a start yourself, I will help you. If you don't do anything, I'm gone. And that hit me like a, er, thunderbolt, knowing that, knowing I, that if I'm not going to help myself, no one else will help me. And that was the beginning, really, of my recovery. (B02)<br><br>Everything I do is like an attempt to rebuild my life but it's taken a long time. (B05)<br><br>Maybe sometimes we need to go through that process, in order to think, I don't need to go back there, to where I was ten years ago, thinking about that experience anymore. But you can think about it, but have a different sort of attitude to it. (B06)<br><br>I tend to be living the moment, that's part of my new self, this post-near death experience self is to really be living in the moment as much as possible, not worry about the future or churn over the past. (D04)<br><br>This was like taking a step back and looking at almost re-engineering life to take into account self-care, self-preservation and also building myself up rather than saying rotten

For peer review only

		stuff about myself and focusing on what I couldn't do and what I should do and should be able to do and all of that. (D08)
**2. Sense of self**	**Development of a more positive sense of self, including integration and valuing of illness experiences**	
2.1 Pride in self	Taking pride in oneself, including personal strengths and achievements	I feel quite proud of my stability. (A01)<br><br>I'm proud of that and that makes me feel better and enables me to operate…I've worked on myself throughout my life and I'm quite proud of that. (A12)<br><br>I believe in my self-worth these days…I must pat myself on the back. (A15)<br><br>Doing well, embracing who you are, loving who you are. Because I went through a long period of neglecting myself, my identity, my race, my culture. (B08)<br><br>I think that's something I'm pretty proud of actually, that I just take people as they are. (B21)<br><br>So yeah, I'm proud of where I've come, do you know what I mean, how far I've come, and that's what help, holds me from stopping and falling apart completely when I think about my daughter, and I get real mad sometimes…That knowing what I've got, and how I've achieved, and I don't want to lose it, cos when she comes to find me I want her to see that I've got these things, and that I'm not a complete arsehole living in a hostel, know what I mean? (C10)
2.2 Integration of experiences	Illness experiences become an accepted part of one's sense of self	That stuff has happened in my life and that is just how it has turned out. Um yeah, that is unfortunate but it is not going to define everything about me. (A07)

For peer review only

		If there was a big red button on the table and somebody said would you like to switch off your diagnosis I would say no because I have accepted it now. Although it was horrid, it was horrible, I can't take anything away from that, but I have accepted that it has become part of my personality and I am happy with that and that's me. (A21)<br><br>I think for a long time I was just existing with the mental health, getting to know, getting to understand it…Because there's very much negativity around it and I felt ashamed. (B06)<br><br>You don't choose the issues that you've got but you can, you can make a choice to change. (C04)<br><br>I am who I am because of what happened. (D04)<br><br>The more I was able to make sense through telling my story of what had happened to me, it then started to have a meaning. (D11)
2.3 Valuing of experiences	Finding positives in the experience of illness	I've had all these amazing experiences and you are just saying it's illness, I just didn't believe it…I feel that it's important and the messages I've got from being in an alternative reality have been extremely important. (A10)<br><br>What they were calling symptoms that must be eradicated, were actually part of me and so I looked behind that and said that is where, that is where my creativity comes from. This is where, this is how I am able to take a photograph, this is how I am able, when I am sitting at a piano to think of a tune. (A19)<br><br>Whereas before I felt ashamed to have these mental health issues, I now feel really proud in a way because I know that I can use it for good and I can make a difference in my community. (B01)<br><br>There is a liberation that comes through all of this, a personal kind of yeah, not sure it's full blown self-actualisation, but it's somewhere up close to the top of the pyramid. (D04)<br><br>I am still me but I am a different me and I am stronger. (D05)

For peer review only

		I kind of thought that that was the best I could ever hope for and that was fine because I kind of really internalized my label of I am an anorexic, I am a you know a broken person who needs a lot of support and actually it was seeing the people who were talking about their lived experience with lots and lots of confidence and taking lots of responsibility and um being empowered to, to self-manage and use their difficulties in a really positive way which kind of opened my eyes to like gosh maybe I am more than my label, maybe I am, maybe I could do more than just function or survive, maybe I could thrive. (D014)
**3. Life perspective**	**New or renewed appreciation of or gratitude about aspects of life**	
3.1 Appreciation of life	Appreciation for life and the importance of hopefulness	I suppose I've become one of those ridiculously, someone I thought I'd never be, I'm becoming one of those ridiculously ever hopeful, ever optimistic people who say there is hope, my life is a life that is about hope, I hope. (A08)<br><br>I have kind of realised that I am not in a position to dictate the terms of life: “it should be like this and I expect this and why is it not like this”. Just, I am alive, I appreciate that I am alive and that I have got so many hours each day to gain something from, some joy or some inspiration or blessings and gifts everywhere that I can and the number one thing is to just keep going, I am a mother and I have to keep going. (C19)
3.2 Appreciation of support	Gratitude for support received from services	So it [organisation] completely changed my view of life. And it's a, it's a roller coaster, it's not like every day's joyous, things would dip, challenges still come in but I deal with them completely differently. Yeah it's like I've found happiness that I never knew existed. (A11)<br><br>Rehab and coming to <service name 1> changed my life, it's like <worker> got me on a college course, it has been absolutely wonderful, it really has, I did a theatre group … and through the rehab I got told about [inaudible] and I came down here <service name 2> and this place is amazing, the things they do here for anybody involved in sex work is absolutely amazing. (C18)
3.3 Meaningful suffering	Gratitude that suffering was	Wow I see where I am and I go back to then and I just think 'you didn't go through that in vain'. (A17)

For peer review only

	meaningful and not in vain	So I think I'm, I suppose I am grateful, for want of another way of putting it, that I have lived the life I have, I have had these experiences. (D04)<br><br>And it's okay, because I wouldn't be where I was, had I not had the experiences that I've had. I don't know what sort of person I'd be, but I certainly wouldn't be where I, doing what I'm doing now. (D12)
3.4 Survivor mission	New growth of political consciousness or use of illness experiences to benefit others	<Organisation> is about re-framing mental illness as a possible catalyst for possible transformation, so and the aim is to give hope to as many people as possible through the power of story-sharing, so I'm really grateful and passionate about this. (A11)<br><br>I just see myself as hopefully being a beacon to others who are, you know, struggling, and others who are finding things difficult. And they can, you know, refer to me [inaudible] support person and they can look at me and think ‘He can make it through all the things that happened to him, then I can make it too’. (B02)<br><br>I think it’s really important to give back to society. And I think that’s what I’m doing through my work. This new chapter I’m going to start, with women now. (B06)<br><br>And so, you know, I felt like I was becoming something. Erm, I could identify with more, kind of, politicised…the personal was political and I was beginning to become aware of that on a deeper level. So, I thought right, that’s it and, I’m going to set up my own thing. (B09)<br><br>And then as I came back out it was just like no, I wanted to help, I wanted to do something, I wanted to use this story, I wanted to actually like you know... I wanted, that's when I started studying psychology and then I applied to go study in <place>…what's helped me recover was I find life, you know, I've got stuff I want to do, I want to help people, I want to, I want to do psychology. (B25)<br><br>I've been looking forward to sharing my experience because um, by people sharing theirs has really helped me and you know, and that makes it feel like talking about it is a

For peer review only

		really worthwhile thing to do, because its gonna allow somebody else to grow, or just take a little bit from it that you know, it might benefit them or it might not, maybe they need to hear a different story but, you know, somebody might, you know, might hear it and think well I'm really pleased that I've heard that because that's, that's gonna help me. (C03)<br><br>I guess in my recovery I tried to be that person for other people, the thing that I didn't have. (C04)<br><br>And my journey through prostitution, uh so I can talk to people about it now. And that's what I want to do, I want to go on and help people if I can that have been through the same thing. (C18)
**4. Wellbeing**	**More active engagement in, and management of, one's own wellbeing and lifestyle**	
4.1 Motivation	Increased determined to stay well, self-manage and not return to a bad situation	The violence started again and I had two broken jaws, a broken nose, scars on my head and scars on my body, but the biggest scar was in my heart and in my mind. I ended up... being put into a secure unit this time and I was meant to be there for three months. It ended up being two weeks, becoming a voluntary patient for a year. Touch wood and whistle, I swore from that day on, no man, money, love or beast would ever put me back into that situation again and I have stuck to it. (A15)<br><br>I started to take my medication, I got rid of this awful man in my life who was my daughter's father. Which was very liberating and, suddenly had an epiphany through all the craziness and thought, I don't want this in my life anymore. If I get rid of certain things it will decrease some of the stress. (B06)

For peer review only

		I have been that low. And that ill, I have been there and I have seen the light and…I have come back from it. I have actually come out of it, I've been there, right there to the lowest of the low. And pulled myself back from it. And that's difficult to do. (C12)<br><br>When I came out of hospital I was like "I can't go back there again so I've got to sort myself out and I've got to build a better relationship with me and the girls", you know, they'd been through so much. (D05)<br><br>Rather than just aimlessly sort of treading water and doing things that I felt I was sort of having to do or other people were you know were saying I should do you know sort of planning about things that actually I enjoyed. (D13)
4.2 Being active	More engagement in the arts, music, sport, nature and learning	I think the art has given me great kind of, great kind of structure. (A01)<br><br>I didn't go into that dangerous space again but I did meet a lot of like-minded people and I played my violin a lot and I decided I wanted to do something with my violin…I would get like a kind of vision of what my purpose was, what my true purpose was in my life and, it was like you know, playing the violin, teaching the violin, it was, which I hadn't been doing because I was working in admin but it just felt really strongly, that is what, it wasn't to do with competition it just felt like, that's the right direction for me to go in and I need to go more and more in that direction, it felt like, like it was connecting me to my soul purpose somehow. (A10)<br><br>I am someone that struggles with relaxing. So, um, gym, I find that I am someone who has to go to the gym, I have to exercise, um and as ridiculous as it sounds, eating as well. You know like I am someone that needs to treat myself good. In order to be good in myself. (A14)<br><br>So that was quite liberating and I didn't think that it would help my mental health without even realising, but I think it did, I did become a bit obsessed with jogging because it was something, I guess, a bit of a break from my children and the monotony of life and the difficult relationship I was in, and the difficulties I had. (B06)

		And erm, and I believe that I need to give more time, a bit more time to, to interests. You know, like, er, I love nature. Absolutely adore nature, it's one of the places I've always felt safe, I could go and sleep in forests, more than I could go and sleep in someone's house. (B09)<br><br>I've been reading quite a lot and educating myself on schizo-affective disorder, plus all the things I've learned about meditation and that kind of thing I practice daily, and I've just found learning to be so rewarding and things, learning through recovery college as well, even though they do suggest reading up on your condition and that kind of thing. So I've just found learning to be so therapeutic and rewarding that I feel like I am at a point now where I can actually study and put my mind towards…doing something worthwhile. (B10)<br><br>I play a lot of music, music is like my, it's my salvation. (B25)<br><br>I could see my body changing and I felt stronger and I felt better and I stopped drinking for a month and I just felt amazing! (D05)<br><br>It was also about sort of about finding activities that I enjoyed much more, rather than just aimlessly sort of treading water and doing things that I felt I was sort of having to do or other people were you know were saying I should do, you know, sort of planning about things that actually I enjoyed… One of the things I did was I joined the choir and you know it's one of [inaudible] gospel choir in South London for about, ever since I got there really and it's a very, very supportive environment, so that's been really critical and I've been able to sort of go there when I've been in every kind of mood state possible. (D13)
**5. Relationships**	**More actively choosing and valuing relationships with others**	

For peer review only

5.1 Choosing relationships	Actively choosing relationships to continue, to re-start or to end	He (counsellor) used a waiter analogy where the waiter is trying to please the clients but then more and more people come in and he is still trying to please them all but the quality of the food comes down and then people start complaining and then it is mad, so basically just concentrate on what you can do and don't try to please everybody…I also now try to mix with people who are just happy and positive and try to stay away from people who aren't. (A09)<br><br>But you need the right quality of people around you, network, for you to be able to stay in your mental health, going forward. (B02)<br><br>I got rid of this awful man in my life. (B06)<br><br>I needed to go back to a couple of my old primary school friends' houses and ask for their forgiveness for something. (B07)<br><br>I'd also come to realise before then that the relationship I was in was not quite what it was as he told me lots of things that were untrue that I discovered so I ended that as well and that was having a clean slate but it took me a long time to get literally over it. (B17)
5.2 Valuing relationships	Placing more value on relationships with others	My children they come at the weekend I give them 100%. I give them 100% because I want to be giving them 100%. (A01)<br><br>But then in another sense it's brought the incredible closeness with people with mental health with my, with my immediate family and friends so it has different angles. (A03)<br><br>It's been a process of learning that I needed, I need desperately, I desperately needed family, you know, people I feel safe with, to be myself. And I needed people to help me normalise my feelings rather than be freaked out and hiding and terrified of expressing. (B09)<br><br>Now I do feel a lot more stable than I did back then, and then when I go to these kinds of experiences you know, I've started to like, connections have become my thing because connections are what saved me from like, connections with people, a spiritual connection

For peer review only

		and this connection between people, there's interconnection between people is essentially what saves people…That power of coming together is so powerful, that is worth fighting for, worth dying for, it's worth me giving my time for. (B25)<br><br>Having the friendship, the respect that built up over that in learning from each other, you know educated, talented, intelligent people. (C04)
5.3 Empathy	Enhanced ability to empathise with others	I can empathise and I do empathise and I use empathy in my work a lot. (A12)<br><br>So my purpose really is young people and even when I see the destruction that young people are going through I never blame them, I said it stems from somewhere…I knew what I went through as a young girl. I could have been a prostitute, I could have been on drugs, I didn't. Do you see what I'm saying? So therefore it's deep. (A17)<br><br>You have understanding, empathy…You really empathise. (B15)
**6. Spirituality**	**Deeper engagement with spirituality, religious and existential endeavours**	
6.1 Spiritual awareness	Increased awareness of the presence of something greater than oneself making a positive contribution by providing meaning	Although the experiences were horrendous, and they were horrendous, there's no other way of describing the agony, it has been necessary because I couldn't do it on my own, so it still feels, and maybe this is just you know the spiritual aspect of my life but it still feels as though I do have like something watches over me and is helping me um and I survived. (A08)<br><br>I kind of relived stuff and at the same time just physically I wanted to let go of loads of things, I just kind of clutter cleared my whole house, really cleaned it, I got rid of loads of bits and pieces that I didn't need any more and as I went through that whole process it was like this massive opening, just kind of spiritual opening again…I felt very very free, I felt like I was being guided with everything, I felt very, very spiritual. Um lots of things happened that were very healing I suppose, played a lot of music…when I relived it and

		the emotion that was associated my voice really did, like it changed, it kind of opened out and it became much more free and liberated and um at the same time I felt like I went into a completely different reality, it was really incredible, it was like intensely spiritual and a feeling that everything was connected and it was though lots of chakras opened or something happened in my body and my mind. (A10)<br><br>Now I'm sort of growing older I know they're spirit animals, I still have them and so it's the wolves that are the most powerful so I do feel protected by those. Um and the other one is [inaudible] spiritual as well I always used to feel some [inaudible] egocentric, sort of like, wounds, [inaudible] kind of reaching out to people because I've always felt like I'm [inaudible] ever since I was really little which has why I have gone into mental health, to help people. (A18)<br><br>But I think the longer deal is this, this, internal shift, they call it a psychic shift, an internal psychic shift, which is more about that spiritual experience and I don't know anything else that, that would work with me, do you know what I mean. And so, erm, since about 2009, that's what I've been kind of exploring in a, in a sort of ad hoc way. (B04)<br><br>But when you kind of realise that there is something bigger than you, you start to realise don't hurt yourself, have more respect for yourself, and you also don't harm others in a sense, not but everything falls into place. The world has meaning, has greater meaning. (C19)
6.2 Spiritual engagement	New or renewed engagement with spiritual or religious practices, helping with meaning-making and providing comfort and security	Like wow so I ran over to them and they kind of just guided me so I was just being guided by all of the spirits and stuff and they guided me up the hill into the woods. (A02)<br><br>…was actually, uh, around all of this I was sort of meditating and looking at the more spiritual aspects of my life and you know, just looking for, I was looking and seeking that help and I was, I was meditating. It, it, it brought a lot of pain but I was looking at that. (A08)

For peer review only

		I became very um sort of what's the word, like spiritual as well. I found the church very comforting and kind of a place I would be, to trust and yeah, it just gave a sense of security for some reason. (A14)<br><br>So, you know, if it wasn't, if it wasn't for yoga, my Buddhist practice and community, who get, who don't, who, who see that there's something valuable from raw emotion, that there's energy in raw emotion…My understanding of Buddhism had, had grown. Erm, I'd been introduced to it, to the practice of chanting [inaudible], to reveal your own courage and compassion and wisdom and create, create more humane connections with people. And, I thought it was amazing energy to, to nourish, 'cos it wasn't based on any gods or idols or who was, you know, who was, who was cool and who wasn't. It's universal. It's a universal truth….the long and short of it, Buddhist practice helped [laughs]. More harmonious with myself and other people. (B09).<br><br>Every morning I put my hand on my heart and I pray and I meditate and I go into that space where it's myself…Now I feel like now I do feel a lot more stable than I did back then and then when I go to these kinds of experiences you know, I've started to like connections have become my thing because connections are what saved me from like, connections with people, a spiritual connection and this connection between people, there's interconnection between people is essentially what saves people. (B25)<br><br>I was still a Quaker at this point so that was really helpful, so I would go each week to have silent worship and that was really good, I saw it as an opportunity to kind of pause and have my brain calm down and yeah that was really lovely. (C15)<br><br>…think also a lot, mental health is to do with emotion, for me I can only find the emotional vocabulary within church. And many things that we are struggling with are deep spiritual psychic rooms and you can't find that power and that passion in a, in anything other than a spiritual context… So for me recovery is about spirituality, it is about God, that is me and that's what works for me, it might not be the same for everyone…just pray, pray. (laughs) It works, do it, don't be scared. (C19)

For peer review only

SRQR Checklist

No.	Topic	Item	Location in text
	Title and abstract		
S1	Title	Concise description of the nature and topic of the study Identifying the study as qualitative or indicating the approach (e.g., ethnography, grounded theory) or data collection methods (e.g., interview, focus group) is recommended	Title
S2	Abstract	Summary of key elements of the study using the abstract format of the intended publication; typically includes background, purpose, methods, results, and conclusions	Abstract
	Introduction		
S3	Problem formulation	Description and significance of the problem/phenomenon studied; review of relevant theory and empirical work; problem statement	Introduction
S4	Purpose or research question	Purpose of the study and specific objectives or questions	Introduction last paragraph
	Methods		
S5	Qualitative approach and research paradigm	Qualitative approach (e.g., ethnography, grounded theory, case study, phenomenology, narrative research) and guiding theory if appropriate; identifying the research paradigm (e.g., postpositivist, constructivist/interpretivist) is also recommended	Methods Procedures paragraph 2
S6	Researcher characteristics and reflexivity	Researchers' characteristics that may influence the research, including personal attributes, qualifications / experience, relationship with participants, assumptions, and/or presuppositions; potential or actual interaction between researchers' characteristics and the research questions, approach, methods, results, and/or transferability	Methods Procedures and Methods Analysis
S7	Context	Setting/site and salient	Methods Setting

For peer review only

		contextual factors	
S8	Sampling strategy	How and why research participants, documents, or events were selected; criteria for deciding when no further sampling was necessary (e.g., sampling saturation); rationale	Methods Setting
S9	Ethical issues pertaining to human subjects	Documentation of approval by an appropriate ethics review board and participant consent, or explanation for lack thereof; other confidentiality and data security issues	Methods paragraph 1
S10	Data collection methods	Types of data collected; details of data collection procedures including (as appropriate) start and stop dates of data collection and analysis, iterative process, triangulation of sources/methods, and modification of procedures in response to evolving study findings	Methods Procedures and Online Supplement 2
S11	Data collection instruments and technologies	Description of instruments (e.g., interview guides, questionnaires) and devices (e.g., audio recorders) used for data collection; if/how the instrument(s) changed over the course of the study	Online Supplement 2
S12	Units of study	Number and relevant characteristics of participants, documents, or events included in the study; level of participation (could be reported in results)	Table 1
S13	Data processing	Methods for processing data prior to and during analysis, including transcription, data entry, data management and security, verification of data integrity, data coding, and anonymization/deidentification of excerpts	Methods Procedures last paragraph
S14	Data analysis	Process by which inferences, themes, etc., were identified and developed, including the researchers involved in data analysis; usually references a specific paradigm or approach; rationale	Methods Analysis and Online Supplement 1 and 3

For peer review only

S15	Techniques to enhance trustworthiness	Techniques to enhance trustworthiness and credibility of data analysis (e.g., member checking, audit trail, triangulation); rationale	Methods Analysis
	Results/findings		
S16	Synthesis and interpretation	Main findings (e.g., interpretations, inferences, and themes); might include development of a theory or model, or integration with prior research or theory	Results Major themes
S17	Links to empirical data	Evidence (e.g., quotes, field notes, text excerpts, photographs) to substantiate analytic findings	Results Category descriptions and Online Supplement 4
	Discussion		
S18	Integration with prior work, implications, transferability, and contribution(s) to the field	Short summary of main findings; explanation of how findings and conclusions connect to, support, elaborate on, or challenge conclusions of earlier scholarship; discussion of scope of application/generalizability; identification of unique contribution(s) to scholarship in a discipline or field	Discussion
S19	Limitations	Trustworthiness and limitations of findings	Discussion paragraph 2
	Other		
S20	Conflicts of interest	Potential sources of influence or perceived influence on study conduct and conclusions; how these were managed	Competing Interests statement
S21	Funding	Sources of funding and other support; role of funders in data collection, interpretation, and reporting	Funding statement

For peer review only

BMJ Open

Post-traumatic growth in mental health recovery: qualitative study of narratives

Journal:	*BMJ Open*
Manuscript ID	bmjopen-2019-029342.R1
Article Type:	Research
Date Submitted by the Author:	12-Apr-2019
Complete List of Authors:	Slade, Mike; University of Nottingham School of Health Sciences,<br>Rennick-Egglestone, Stefan; University of Nottingham School of Health Sciences, Institute of Mental Health<br>Blackie, Laura; University of Nottingham, Department of Psychology<br>Llewellyn-Beardsley, Joy; University of Nottingham School of Health Sciences, Institute of Mental Health<br>Franklin, Donna; NEON Lived Experience Advisory Panel<br>Hui, Ada; University of Nottingham School of Health Sciences<br>Thornicroft, Graham; institute of psychiatry, health service and population research<br>McGranahan, Rose; Queen Mary University of London, Unit of Social and Community Psychiatry<br>Pollock, Kristian; Nottingham University, School of Nursing<br>Priebe, Stefan; Barts and the London School of Medicine and Dentistry, University of London, Unit of Social and Community Psychiatry<br>Ramsay, Amy; Institute of Psychiatry, Psychology and Neuroscience, King's College London<br>Roe, David; University of Haifa, Department of Community Mental Health<br>Deakin, Emilia; University of Nottingham School of Health Sciences, Institute of Mental Health
**Primary Subject Heading**:	Mental health
Secondary Subject Heading:	Qualitative research
Keywords:	Post-traumatic growth, MENTAL HEALTH, recovery

SCHOLARONE™
Manuscripts

Post-traumatic growth in mental health recovery: qualitative study of narratives

Mike Slade[1*]
Stefan Rennick-Egglestone[1]
Laura E. R. Blackie[2]
Joy Llewellyn-Beardsley[1]
Donna Franklin[3]
Ada Hui[1]
Graham Thornicroft[4]
Rose McGranahan[5]
Kristian Pollock[6]
Stefan Priebe[5]
Amy Ramsay[4]
David Roe[7]
Emilia Deakin[1]

[1] School of Health Sciences, Institute of Mental Health, University of Nottingham, UK
[2] School of Psychology, University of Nottingham, UK
[3] NEON Lived Experience Advisory Panel, Nottingham, UK
[4] Centre for Implementation Science, Health Service and Population Research Department, Institute of Psychiatry, Psychology and Neuroscience, King's College London, UK
[5] Unit of Social and Community Psychiatry, Queen Mary University of London
[6] School of Health Sciences, University of Nottingham, UK
[7] Department of Community Mental Health, University of Haifa, Israel

[*] Corresponding author
Email: m.slade@nottingham.ac.uk
Address: School of Health Sciences, Institute of Mental Health, University of Nottingham, Triumph Road, Nottingham NG7 2TU, UK

For peer review only - http://bmjopen.bmj.com/site/about/guidelines.xhtml

Abstract

Objectives

Post-traumatic growth, defined as positive psychological change experienced as a result of the struggle with challenging life circumstances, is under-researched in people with mental health problems. The aim of this study was to develop a conceptual framework for post-traumatic growth in the context of recovery for people with psychosis and other severe mental health problems.

Design

Qualitative thematic analysis of cross-sectional semi-structured interviews about personal experiences of mental health recovery.

Setting

England.

Participants

Participants were adults aged over 18 and: (i) living with psychosis and not using mental health services (n=21); (ii) using mental health services and from black and minority ethnic communities (n=21); (iii) underserved, operationalised as lesbian, gay, bisexual and transgender community or complex needs or rural community (n=19); or (iv) employed in peer roles using their lived experience with others (n=16). The 77 participants comprised 42 (55%) female and 44 (57%) White British.

Results

Components of post-traumatic growth were present in 64 (83%) of recovery narratives. Six superordinate categories were identified, consistent with a view that post-traumatic growth involves learning about oneself (Self-discovery) leading to a new sense of who one is (Sense of self) and appreciation of life (Life perspective). Observable positively-valued changes comprise a greater focus on self-management (Wellbeing) and more

For peer review only - http://bmjopen.bmj.com/site/about/guidelines.xhtml

importance being attached to relationships (Relationships) and spiritual or religious engagement (Spirituality). Categories are non-ordered and individuals may start from any point in this process.

Conclusions

Post-traumatic growth is often part of mental health recovery. Changes are compatible with research about growth following trauma, but with more emphasis on self-discovery, integration of illness-related experiences and active self-management of wellbeing. Trauma-related growth may be a preferable term for participants who identify as having experienced trauma. Trauma-informed mental health care could use the six identified categories as a basis for new approaches to supporting recovery.

Key words

Post-traumatic growth, mental health, recovery

Trial registration

Conducted as part of the NIHR NEON Study (ISRCTN11152837).

For peer review only - http://bmjopen.bmj.com/site/about/guidelines.xhtml

Article summary

Strengths and limitations of this study

- This study reports findings from a substantial qualitative investigation of post-traumatic growth in people with psychosis and other severe mental health problems
- The purposive sampling involved under-researched groups, including people with mental health problems who do not use mental health services or who are underserved by mental health services
- The involvement of eight analysts from varied backgrounds including several with lived experience of mental ill-health increases the credibility of the data analysis
- Participants were self-selecting so the views of people who do not associate with the term 'recovery' may be under-represented
- Participants were not asked specifically about trauma experiences

For peer review only - http://bmjopen.bmj.com/site/about/guidelines.xhtml

Introduction

Post-traumatic growth is a relatively new area of research. The concept was introduced twenty years ago,[1] and is defined as perceptions of 'positive psychological change experienced as a result of the struggle with highly challenging life circumstances'.[2] Other terms for this phenomenon have also been used, such as benefit-finding,[3] both a coping profile and a coping outcome,[4] and positive illusions.[5] This range of terms points to the complexity of the phenomenon, with different theorists emphasising change in identity and narrative (i.e. the sense of self),[6] change in eudaimonic wellbeing (i.e. subjective quality of life),[7] and change in social/psychological resources (i.e. broadening response repertoires).[8] However, there is broad consensus over five post-traumatic growth domains: improved relations with others; identification of new possibilities for one's life; increased perception of personal strengths; spiritual growth; and enhanced appreciation of life.[2]

Post-traumatic growth is now a well-established concept in relation to 'event trauma', i.e. experience of a single traumatic event. Post-traumatic growth is widely reported in relation to event trauma and it is associated with mental health outcomes, such as depression. However, the direction of relationships between PTG and depression are not consistent across studies. For example, post-traumatic growth was found to moderate the negative association with quality of life found for both depression and post-traumatic stress in 58 Norwegian survivors interviewed two and six years after the 2004 Southeast Asia tsunami.[9] Other studies have shown that post-traumatic growth is associated with a higher level of depression, such as a two-year follow-up study of 316 survivors of the L'Aquila earthquake in Italy.[10] Similarly, higher post-traumatic growth was associated with lower post-traumatic stress disorder and higher

For peer review only - http://bmjopen.bmj.com/site/about/guidelines.xhtml

depression in 186 Iraqi students with an average of 13 war-related adversity experiences.[11] There is some evidence of a curvilinear association between depression and post-traumatic growth, for example two studies of assault survivors (n=270 in total) found survivors with low or high post-traumatic growth reported more symptoms of post-traumatic stress (both studies) and depression (one study) than those with intermediate growth levels.[12] Longitudinal research is needed to fully understand how reports of post-traumatic growth interact with depression in the recovery from event trauma.

Post-traumatic growth has relevance to health care, for example liver transplantation[13] and stroke.[14] There are several reasons why post-traumatic growth may also be relevant to psychosis and other severe mental health problems.[15] First, abuse incidence is high. In particular, childhood adversity is strongly associated with increased risk of psychosis. A meta-analysis of 18 case-control studies (n=2,048 psychosis, n=1,856 controls), 10 prospective studies (n=41,803) and 8 population-based cross-sectional studies (n=35,546) found significant associations between adversity and psychosis across all designs (OR2.78, 95%CI 2.34–3.31).[16] Second, comorbid depression, which is implicated in post-traumatic growth, is common. For example, around 50% of people with a diagnosis of schizophrenia also experience depression.[17] Third, both the experience of psychosis itself and the consequent experiences of societal discrimination and re-traumatisation caused by mental health system responses[18,19] may generate trauma. Finally, some people with personal experience of psychosis report post-traumatic growth.[20,21]

For peer review only - http://bmjopen.bmj.com/site/about/guidelines.xhtml

There is strong evidence that positive changes can be experienced after first-episode psychosis. A systematic review,[22] published initially as a scoping review,[23] identified 40 studies involving 715 participants investigating the experience of positive change after first episode psychosis. The review identified three levels of positive change. The individual-level change theme was developed by combining three sub-themes: insight and clarity (e.g. reassessing one's life, realising who one's friends are, less emphasis on materialism and societal expectations); personality, outlook and skills (e.g. more authenticity, better able to handle stress, learning time management); and health, lifestyle and interests (e.g. simpler life, new possibilities, better sleep habits). The interpersonal-level change theme was developed by combining two sub-themes: relationships with family and friends (improvements in communication, spending more time together, letting go of unhealthy relationships) and place or role in society (desiring to give back, challenging stigma in society). The religious or spiritual-level theme examples were praying more, increased engagement in religious institutions and positive religious experiences.

There is only limited evidence about the frequency and types of post-traumatic growth in people living long-term with psychosis and other severe mental illness experiences.[24] A quantitative study of 121 people with severe mental illness using community mental health rehabilitation centres in Israel found high levels of trauma,[25] and that meaning-making and coping self-efficacy mediated post-traumatic growth experiences.[26] Three small (n=7,[27] n=7,[28] n=10[29]) qualitative studies using interpretative phenomenological analysis of semi-structured interviews all identified themes of personal growth.

For peer review only - http://bmjopen.bmj.com/site/about/guidelines.xhtml

The concept of recovery in mental health has come to mean living as well as possible, whether or not symptoms are present. Recovery thus differs from the traditional clinical priorities of symptom remission and functional restoration,[30] and a systematic review identified five recovery processes: Connectedness, Hope, Identity, Meaning and Empowerment (CHIME Framework).[31] Current evidence indicates that growth is integral to recovery, and involves both restoration of existing aspects of identity and construction of new aspects.[32]

The extent to which (a) the five growth processes identified from event trauma research and (b) the more preliminary early psychosis-specific restorative and constructive identity processes are characterising the same changes is unknown. Integration of these two sources of evidence is needed, as is investigation of the experiences of a broader range of people with long-term psychosis, including under-researched groups. The aim of this study is to develop a conceptual framework for post-traumatic growth in the context of recovery for people with psychosis and other severe mental health problems.

Methods

This research was undertaken as part of the NIHR Narrative Experiences Online (NEON) study (ISRCTN11152837, information at http://www.researchintorecovery.com/neon) between March and August 2018. Ethical Committee approval was obtained (Nottingham 2 REC 17/EM/0401). All participants provided written informed consent.

Participants

For peer review only - http://bmjopen.bmj.com/site/about/guidelines.xhtml

A purposive sample of under-researched populations took part. Inclusion criteria common to all groups were: aged over 18; willing to discuss experiences; able to give informed consent; and fluent in English.

Group A (Outside the system) comprised people having had self-identified experiences of psychosis of sufficient frequency or duration that they identify as someone with experience of psychosis, and not using services. Psychosis experience prevalence estimates in the general population range from 7%[33] to 13%,[34] yet lifetime rates of psychosis, determined through contact with services, range from 0.2% (narrowly defined criteria) to 0.7% (broadly defined).[35] The experience of the many individuals who have psychosis without using services may therefore illuminate growth processes. Additional inclusion criteria for Group A were: self-identified lifetime experience of psychosis; no use of secondary mental health services over the previous five years.

Group B (BAME) comprised people who identified as being from Black and Minority Ethnic (BAME) populations. Ethnic minority groups often have problematic relationships with services,[36] and research about recovery in these populations identifies a strong emphasis on the post-traumatic growth concepts of connectedness[31] and spirituality.[37] Additional inclusion criteria for Group B were: currently using mental health services; Black, Asian and minority ethnic community member.

Group C (Under-served) comprised people who were not well engaged with mental health services. This was operationalised for three under-served groups: people from

For peer review only - http://bmjopen.bmj.com/site/about/guidelines.xhtml

lesbian, gay, bisexual or trans (LGBT+) communities;[38] from rural communities;[39] or with multiple complex health and social care needs.[40] Additional inclusion criteria for Group C were: experience of mental health problems in previous 10 years; no or mainly unsuccessful interactions with formal mental health services; member of LGBT+ communities OR living in an electoral district area with less than 10,000 population OR experience of at least two of homelessness, substance misuse issues or offending.

Group D: (Peer) comprised people with experience of working in statutory or voluntary roles for which lived experience is a requirement, e.g. peer support workers, trainers or researchers. Addition inclusion criteria for Group D were: working in statutory or voluntary roles for which lived experience is an essential requirement; use their lived experience as a tool for engagement with other mental health service users.

Setting

Participants were recruited across England, with Groups A (Outside the system) and B (BAME) primarily from London and Groups C (Under-served) and D (Peer) primarily from the Midlands. Guidelines for recruitment are shown in Online Supplement 1. Group A were recruited through primary care services support groups, Hearing Voices Network, and online advertising. Group B were recruited through community groups, a Recovery College and psychosis-specific secondary mental health services. Group C were recruited through community networks, voluntary sector organisations and secondary care mental health services. Group D were recruited through community groups and secondary care mental health services. Recruitment for all groups used snowball sampling.

For peer review only - http://bmjopen.bmj.com/site/about/guidelines.xhtml

Procedures

A preliminary coding framework (Online Supplement 2) was developed in advance of interviews, by collating existing post-traumatic growth dimensions identified in trauma populations[41] and in previous studies of post-traumatic growth in psychosis.[22,23,28] Duplicates were removed, dimension names were made consistent, and then thematic grouping of the dimensions was used to develop the preliminary coding framework, which was intended to establish the link between existing research and participant narratives, and comprised the code name, definition and examples drawn from the source references. An 'Other' category was added to allow the emergence of new themes.

Each participant took part in a 40-90 minute interview conducted in a health service or community venue. Interviews using a narrative approach were conducted by four researchers from sociology, advocacy, public health and health psychology backgrounds. The topic guide (shown in Online Supplement 3) asked the participant to share their mental health and recovery experiences, as if it were a story, with a beginning, a middle and an end, and to include some consideration of what might happen in the future. Participants were remunerated (£20) for their time, and given options to pause or discontinue if they became distressed. Interviews were recorded, and pseudonymised transcripts were made after interviews. After the interview the researcher wrote field notes, which were included in the analysis.

Analysis

For peer review only - http://bmjopen.bmj.com/site/about/guidelines.xhtml

The four coders comprised three interviewers plus one non-interviewer with an interdisciplinary background in sociology and mental health nursing. Thematic analysis was undertaken using NVivo version 11.[42] The study had a predetermined focus on developing a conceptual framework starting from a synthesis of empirical evidence derived from event trauma research as described above, which was then developed through analysis of interview data. An approach akin to framework analysis was adopted[43]. Framework analysis involves applying *a priori* codes and categories to qualitative data to explore specific questions of interest to the research aims, with attention also paid to inductive analysis of data relating to topics which were not anticipated in advance, and to responsive revision of the coding frame as analysis progresses and in the light of regular discussion within the research team.

Coding was initially according to the preliminary coding framework and informed by emergent understandings captured by field notes, but coders remained open to the identification of additional themes in the data. Coding involved identification and allocation of text relating to the coding framework, enabling related text to be grouped and compared, allowing identification of themes occurring within and across sources. Regular discussions between analysts explored how themes of post-traumatic growth were expressed and related to each other, allowing lower order themes to be recognised.[44] Each coder independently coded and compared the same initial transcript. Remaining transcripts were then coded separately (25% per coder).

The coding framework was then iteratively refined in meetings between the four primary coders and a wider group of four other non-interviewer analysts with expertise in healthcare technologies, qualitative research, recovery research and clinical

For peer review only - http://bmjopen.bmj.com/site/about/guidelines.xhtml

psychology. Several of the interviewers, coders and analysts also had lived experience of mental ill-health and recovery, to enhance the role of lived experience in collection and analysis of data.[45] To enhance trustworthiness, an audit trail was kept, and an interim coding framework is shown in Online Supplement 4. The conceptual framework, i.e. the final coding framework, was agreed by all coders and analysts.

Results

The sociodemographic and clinical characteristics of the 77 participants are shown in Table 1.

Insert Table 1 here

Post-traumatic growth components were coded in 64 (83%) of the 77 transcripts. The conceptual framework for post-traumatic growth in psychosis and other severe mental health problems is summarised in Table 2 with a complete version including more example coding in Online Supplement 5.

Insert Table 2 here

Major themes

Theme 1: Self-discovery

The self-discovery theme involves a fuller and deeper understanding of oneself. This involves the ability to access, accept and be mindful of difficult feelings:

…what was going on was an internal not an external thing. (B04)

…the key to everything isn't it, accessing your emotions, not running away from them… (C19)

The resulting self-knowledge leads to greater authenticity and being less influenced by the expectations of others

I feel like I know myself quite well, you know I can heal myself. (A08)

When I discovered that freedom, that I didn't have to join the rat-race…that was quite liberating… (B18)

Self-acceptance can arise from greater self-knowledge, by letting go of past difficulties and developing self-compassion

It's all about self-accepting, getting to know me and it helped. (A17)

The key word is accepting the situation that I was in, um, and being honest with myself. (C03)

Alongside these processes, participants talked about the importance of taking, or taking back, responsibility for one's own life

For peer review only - http://bmjopen.bmj.com/site/about/guidelines.xhtml

> *…that hit me like a, er, thunderbolt, knowing that, knowing I, that if I'm not going to help myself, no one else will help me. And that was the beginning, really, of my recovery.* (B02)

> *This was like taking a step back and looking at almost re-engineering life to take into account self-care, self-preservation and also building myself up…* (D08)

Theme 2: Sense of self

The development of a more positive sense of self involved integration and valuing of illness experiences. A repeated theme was pride in oneself as a person.

> *I believe in my self-worth these days…I must pat myself on the back.* (A15)

> *I think that's something I'm pretty proud of actually, that I just take people as they are.* (B21)

An important part of this pride was integrating experiences of mental ill-health so they become an accepted part of one's sense of self.

> *You don't choose the issues that you've got but you can, you can make a choice to change.* (C04)

> *I am who I am because of what happened.* (D04)

For peer review only - http://bmjopen.bmj.com/site/about/guidelines.xhtml

Some participants moved beyond accepting to positively valuing these experiences.

> *What they were calling symptoms that must be eradicated, were actually part of me and so I looked behind that and said that is where, that is where my creativity comes from.* (A19)

> *I am still me but I am a different me and I am stronger.* (D05)

Theme 3: Life perspective

Participants identified a new or renewed appreciation of, or gratitude about, aspects of life. For some this was a general sense of appreciation.

> *…I'm becoming one of those ridiculously ever hopeful, ever optimistic people who say there is hope, my life is a life that is about hope...* (A08)

> *…I am alive, I appreciate that I am alive…* (C19)

For others there was a new appreciation of support received from support groups, mental health services and workers.

> *…it [organisation] completely changed my view of life.* (A11)

> *Rehab and coming to <service name> changed my life, it's like <worker> got me on a college course, it has been absolutely wonderful…* (C18)

For peer review only - http://bmjopen.bmj.com/site/about/guidelines.xhtml

The idea that suffering has been meaningful or worthwhile was expressed.

> *Wow I see where I am and I go back to then and I just think 'you didn't go through that in vain'.* (A17)

> *…I suppose I am grateful, for want of another way of putting it, that I have lived the life I have, I have had these experiences.* (D04)

New connections with political consciousness or a survivor mission to ensure others did not have similar bad experiences were identified by some participants, especially in Group B.

> *…I could identify with more, kind of, politicised…the personal was political and I was beginning to become aware of that on a deeper level.* (B09)

> *And then as I came back out it was just like no, I wanted to help…I've got stuff I want to do, I want to help people...* (B25)

Theme 4: Wellbeing

The above psychological processes were complemented by a more active engagement in managing wellbeing and lifestyle. A determination to stay well was identified.

> *…I swore from that day on, no man, money, love or beast would ever put me back into that situation again and I have stuck to it.* (A15)

For peer review only - http://bmjopen.bmj.com/site/about/guidelines.xhtml

…I've been there, right there to the lowest of the low. And pulled myself back from it. And that's difficult to do. (C12)

This led to a greater engagement in wellbeing-related activities, including arts, music, sport, nature and learning

I think the art has given me great kind of, great kind of structure. (A01)

…I've just found learning to be so therapeutic and rewarding that I feel like I am at a point now where I can actually study and put my mind towards…doing something worthwhile. (B10)

Theme 5: Relationships

Many participants identified changes in relationships. For some this involved more actively choosing which relationships to continue, to re-start or to end

I got rid of this awful man in my life. (B06)

I needed to go back to a couple of my old primary school friends' houses and ask for their forgiveness for something. (B07)

For others, the change was in the value placed on relationships with others.

For peer review only - http://bmjopen.bmj.com/site/about/guidelines.xhtml

…it's brought the incredible closeness with people with mental health with my, with my immediate family and friends… (A03)

It's been a process of learning that I needed, I need desperately, I desperately needed family, you know, people I feel safe with, to be myself. (B09)

A greater ability to empathise with others was also identified.

So my purpose really is young people and even when I see the destruction that young people are going through I never blame them, I said it stems from somewhere… (A17)

You have understanding, empathy…You really empathise. (B15)

All of these relationship processes informed a desire to give back, both by supporting others in similar situations and by giving back to society. This code differs from the survivor mission and mutuality sub-themes in its emphasis on giving as intrinsically beneficial.

I just see myself as hopefully being a beacon to others who are, you know, struggling, and others who are finding things difficult. (B02)

I want to go on and help people if I can that have been through the same thing. (C18)

For peer review only - http://bmjopen.bmj.com/site/about/guidelines.xhtml

Theme 6: Spirituality

Participants, especially in Group A, described a deeper engagement with spirituality, religious and existential endeavours. For some, this was expressed as an enhanced spiritual awareness.

> *Now I'm sort of growing older I know they're spirit animals, I still have them and so it's the wolves that are the most powerful so I do feel protected by those.* (A18)

> *…as I went through that whole process it was like this massive opening, just kind of spiritual opening again.* (A10)

This was associated with new or renewed observable engagement in spiritual or religious practices.

> *… I was sort of meditating and looking at the more spiritual aspects of my life and…I was looking and seeking that help…* (A08)

> *…just pray, pray. (laughs) It works, do it, don't be scared.* (C19)

Discussion

Post-traumatic growth concepts were identified in 64 (83%) of the interviews with 77 diverse participants describing their mental health recovery, and no participants rejected the idea of post-traumatic growth. The six superordinate categories are not ordered, but one narrative consistent with the results is that post-traumatic growth

involves learning about oneself (Self-discovery) leading to a new sense of who one is (Sense of self) and one's appreciation of life (Life perspective). Observable positively-valued changes are a greater focus on self-management (Wellbeing) and more importance being attached to relationships (Relationships) and spiritual / religious engagement (Spirituality). Individuals may start from any point in this narrative, so an alternative description would be that individuals experience a change in their Life perspective, enabling an alternative Sense of self to be developed, which facilitates new kinds of Self-Discovery. The conceptual framework is compatible with the five growth processes identified in event trauma research, but participants also experienced changes in (a) self-discovery, (b) sense of self, specifically including integration of illness-related experiences into identity and (c) the importance they attached to active self-management of well-being. Many participants were currently struggling with adversity so the term 'post-traumatic' may need amending, perhaps to 'trauma-related' for those who view their experiences as related to trauma.

The strengths of the study include the relatively large sample size for a qualitative study, the purposive sampling of diverse and under-researched participant groups, and the large analyst team (n=8) bringing multiple perspectives to enhance trustworthiness of data analysis. Weaknesses include the limited geographical spread mainly from two parts of England, the absence of member-checking, not collecting data on historical mental health service use by Group A participants, and an assumption that trauma was present and therefore not asked about in interview (allowing assessment of the extent to which participants framed their experiences as 'trauma'). Identifying a change as positive was not always straightforward, so change experiences which in this study were not coded as positive psychological change may

For peer review only - http://bmjopen.bmj.com/site/about/guidelines.xhtml

merit further investigation. These were not dissenting voices or competing accounts, but statements about types of change which were not judged by analysts to be post-traumatic growth. Examples which were not coded as post traumatic growth include alternative methods of getting well, escapism, knowing where to access support, cultural differences and environmental factors. Recovering from 'trauma' was not the language or frame of reference used by many participants in telling their stories, so this represents a researcher framing of experiences. However, many of the changes identified by participants were clearly describing growth arising directly out of their experiences. Relatedly, the extent to which participants attributed changes to post-traumatic growth, and the extent to which their formulations were derived from cultural understandings, were not explored. Our data are consistent with a view that the development of trauma-informed mental health services, with consequent changes in language and constructs used to describe experiences, will lead to an increase in individuals using post-traumatic growth concepts in their own recovery narratives. An alternative analysis approach would have involved wholly inductive coding followed by comparison with existing research. This would be more applicable to an interview study using a topic guide to assess previous experiences of trauma and identifying specifically post-traumatic growth experiences, rather than the current topic guide which was focussed on recovery narratives and did not specifically ask about previous trauma experiences. The wide diagnostic spread of participants and high proportion (36%) who preferred not to give a diagnosis could be seen as a limitation. The standard health research approach would be to use a standardised assessment to confirm research diagnosis, but recovery research tends to have a more trans-diagnostic focus. Finally, the cross-sectional coding inevitably abstracts from complex

For peer review only - http://bmjopen.bmj.com/site/about/guidelines.xhtml

and contextualised stories, so may not capture the ambivalent and conflicting perspectives common in narratives.

This study provides further evidence that post-traumatic growth concepts may be relevant to many people living with psychosis and other severe mental health problems, including previously under-research groups. A mental health service orientation towards supporting recovery is recommended internationally,[46,47] and central to national policy in many countries.[48-51] A recovery orientation involves system transformation[52] requiring new clinical approaches including a greater emphasis on supporting strengths,[53-55] self-management,[56,57] hope,[58,59] and wellbeing,[60-62] more use of new interventions such as positive psychology,[63,64] Recovery Colleges[65-68] and peer support,[69,70] and a greater focus on human rights.[71,72] The current study supports the case that trauma-informed approaches to mental health care should be added to this list of recovery-supporting innovations.[73]

The conceptual framework provides a theoretical foundation for work to support post-traumatic growth in mental health. Encouraging post-traumatic growth is an important contribution to supporting recovery. For example, a study of post-traumatic growth and recovery in 34 people experiencing first episode psychosis concluded that '*people with early psychosis may benefit from disclosing their experiences of psychosis, including those aspects that were traumatic, as this may support the processes of recovery and post-traumatic growth*' (p.213).[74] A clinical implication is that it is important to reinforce efforts by service users to find their own personally satisfactory meaning of their experiences, rather than simply encouraging the adoption of a clinical explanatory model. Similarly, supporting service users to engage in wellbeing self-management,

For peer review only - http://bmjopen.bmj.com/site/about/guidelines.xhtml

to actively choose relationships and to explore spiritual development should be a significant clinical focus.

The relationship between mental health recovery and post-traumatic growth is unclear, as is the extent to which they are the same or overlapping but distinct phenomena. Prospective longitudinal research is needed to investigate if there is a temporal relationship between positive psychological change and development of an identity as a 'person in recovery' for different clinical sub-populations, and how these change in the context of crisis or relapse. The implications for mental health care processes also need to be investigated, specifically identifying clinical sub-populations for whom a post-traumatic growth approach is particularly indicated, and developing and evaluating manualised treatment approaches which support the identified positive psychological changes. What is clear is that moving forward from severe mental health problems is not simply a question of taking treatment as prescribed, but a far more active process of engaging and re-engaging in the search for meaning for oneself, learning to manage the demands of living well, and finding one's place in the world.

For peer review only - http://bmjopen.bmj.com/site/about/guidelines.xhtml

References

1. Tedeschi R. Violence transformed: posttraumatic growth in survivors and their societies. *Aggressive and Violent Behavior* 1999; **4**: 319-41.
2. Tedeschi R, Calhoun L. Posttraumatic growth: Conceptual foundations and empirical evidence. *Psychological Inquiry* 2004; **15**: 1–18.
3. Tomich P, Helgeson V. Is finding something good in the bad always good? Benefit finding among women with breast cancer. *Health Psychol* 2004; **23**: 16–23.
4. Zoellner T, Maercker A. Posttraumatic growth in clinical psychology - a critical review and introduction of a two component model. *Clin Psychol Rev* 2006; **26**: 626-53.
5. Taylor S, Armor D. Positive illusions and coping with trauma. *J Pers* 1996; **64**: 873–98.
6. Pals J, McAdams D. The transformed self: A narrative understanding of posttraumatic growth. *Psychological Inquiry* 2004; **15**: 65–9.
7. Joseph S, Linley P. Positive adjustment to threatening events: An organismic valuing theory of growth through trauma. *Review of General Psychology* 2005; **9**: 262–80.
8. Hobfoll S, Hall B, Canetti-Nisim D, Galea S, Johnson R, Palmieri P. Refining our understanding of traumatic growth in the face of terrorism: Moving from meaning cognitions to doing what is meaningful. *Applied Psychology: An International Review* 2007; **56**: 345–66.
9. Siqveland J, Nygaard E, Hussain A, Tedeschi R, Heir T. Posttraumatic growth, depression and posttraumatic stress in relation to quality of life in tsunami survivors: a longitudinal study. *Health and Quality of Life Outcomes* 2015; **13**: 18.
10. Bianchini V, Giusti L, Salza A, et al. Moderate Depression Promotes Posttraumatic Growth (Ptg): A Young Population Survey 2 Years after the 2009 L'Aquila Earthquake. *Clinical Practice & Epidemiology in Mental Health* 2017; **13**: 10-9.
11. Magruder M, Kılıç C, Koryürek M. Relationship of posttraumatic growth to symptoms of posttraumatic stress disorder and depression: A pilot study of Iraqi students. *International Journal of Psychology* 2015; **50**: 402-6.
12. Kleim B, Ehlers A. Evidence for a Curvilinear Relationship Between Posttraumatic Growth and Posttrauma Depression and PTSD in Assault Survivors. *J Trauma Stress* 2009; **22**: 45-52.
13. Pérez-San-Gregorio M, Martín-Rodríguez A, Borda-Mas M, et al. Post-traumatic growth and its relationship to quality of life up to 9 years after liver transplantation: a cross-sectional study in Spain. *BMJ Open* 2017; **7**: e017455.
14. Shipley J, Luker J, Thijs V, Bernhardt J. The personal and social experiences of community-dwelling younger adults after stroke in Australia: a qualitative interview study. *BMJ Open* 2018; **8**: e023525.
15. Dunkley J, Bates G, Foulds M, Fitzgerald P. Understanding Adaptation to First-Episode Psychosis: The Relevance of Trauma and Posttraumatic Growth. *Australasian Journal of Disaster and Trauma Studies* 2007; **2007-1**: 1-16.
16. Varese F, Smeets F, Drukker M, et al. Childhood Adversities Increase the Risk of Psychosis: A Meta-analysis of Patient-Control, Prospective- and Cross-sectional Cohort Studies. *Schizophr Bull* 2012; **38**(4): 661-71.
17. Buckley P, Miller, B., Lehrer, D., Castle, D. Psychiatric Comorbidities and Schizophrenia. *Schizophr Bull* 2009; **35**: 383-402.

For peer review only - http://bmjopen.bmj.com/site/about/guidelines.xhtml

18. Bloom S, Farragher B. Destroying Sanctuary: The Crisis in Human Service Delivery Systems. Oxford: Oxford University Press; 2010.
19. Priebe S, Bröker M, Gunkel S. Involuntary Admission and Posttraumatic Stress Disorder Symptoms in Schizophrenia Patients. *Compr Psychiatry* 1998; **39**: 220-4.
20. Chadwick PK. Schizophrenia: The Positive Perspective. London: Routledge; 1997.
21. Roe D, Chopra M. Beyond Coping with Mental Illness: Towards Personal Growth. *Am J Orthopsychiatry* 2003; **73**: 334-44.
22. Jordan G, MacDonald K, Pope M, Schorr E, Malla A, Iyer S. Positive Changes Experienced After a First Episode of Psychosis: A Systematic Review. *Psychiatr Serv* 2018; **69**: 84-99.
23. Jordan G, Pope, M., Lambrou, A., Malla, A., Iyer, S. Post-traumatic growth following a first episode of psychosis: a scoping review. *Early Intervention in Psychiatry* 2017; **11**: 187-99.
24. Slade M, Blackie L, Longden E. Personal growth in psychosis. *World Psychiatry* 2019; **18**: 29-30.
25. Mazor Y, Gelkopf, M., Mueser, K., Roe, D. Posttraumatic growth in psychosis. *Frontiers in Psychiatry* 2016; **7**: 202.
26. Mazor Y, Gelkopf M, Roe D. Posttraumatic growth among people with serious mental illness, psychosis and posttraumatic stress symptoms. *Compr Psychiatry* 2018; **81**: 1-9.
27. Dixon L, Sanderson C, Holt L. A Weird but Interesting Journey: Personal Traumatic Growth for Individuals with Hallucinations. *Journal of Psychology and Psychotherapy* 2018; **8**: 343.
28. Mapplebeck C, Joseph S, Sabin-Farrell R. An Interpretative Phenomenological Analysis of Posttraumatic Growth in People With Psychosis. *Journal of Loss and Trauma* 2015; **20**: 34-45.
29. Attard A, Larkin M, Boden Z, Jackson C. Understanding Adaptation to First Episode Psychosis Through the Creation of Images. *Journal of Psychosocial Rehabilitation and Mental Health* 2017; **4**: 73-88.
30. Palmquist L, Patterson S, O'Donovan A, Bradley G. Protocol: A grounded theory of 'recovery'—perspectives of adolescent users of mental health services. *BMJ Open* 2017; **7**: e015161.
31. Leamy M, Bird V, Le Boutillier C, Williams J, Slade M. A conceptual framework for personal recovery in mental health: systematic review and narrative synthesis. *Br J Psychiatry* 2011; **199**: 445-52.
32. Dunkley J, Bates G. Recovery and adaptation after first-episode psychosis: The relevance of posttraumatic growth. *Psychosis* 2015; **7**: 130-40.
33. Linscott R, van Os J. An updated and conservative systematic review and meta-analysis of epidemiological evidence on psychotic experiences in children and adults: on the pathway from proneness to persistence to dimensional expression across mental disorders. *Psychol Med* 2013; **43**: 1133-49.
34. Beavan V, Read, J., Cartwright, C. The prevalence of voice-hearers in the general population: A literature review. *Journal of Mental Health* 2011; **20**: 281-92.
35. Kendler KS, Gallagher TJ, Abelson JM, Kessler RC. Lifetime prevalence, demographic risk factors, and diagnostic validity of nonaffective psychosis as assessed in a US community sample. The National Comorbidity Survey. *Arch Gen Psychiatry* 1996; **53**: 1022-31.

For peer review only - http://bmjopen.bmj.com/site/about/guidelines.xhtml

36. Ghali S, Fisher HL, Joyce J, et al. Ethnic variations in pathways into early intervention services for psychosis. *Br J Psychiatry* 2013; **202**(4): 277-83.
37. Whitley R. Ethno-Racial Variation in Recovery From Severe Mental Illness: A Qualitative Comparison. *Canadian Journal of Psychiatry* 2016; **61**: 340-7puschner.
38. Hudson-Sharp N, Metcalf H. Inequality among lesbian, gay bisexual and transgender groups in the UK: a review of evidence. London: NIESR, 2016.
39. Gunnell D, Wheeler B, Chang S, Thomas B, Sterne J, Dorling D. Changes in the geography of suicide in young men: England and Wales 1981–2005. *J Epidemiol Community Health* 2012; **66**: 536-43.
40. Kuluski K, Ho J, Hans K, Nelson M. Community Care for People with Complex Care Needs: Bridging the Gap between Health and Social Care. *International Journal of Integrated Care* 2017; **17**: 1-11.
41. Jayawickreme E, Blackie, L. Post-traumatic growth as positive personality change: Evidence, controversies and future directions. *European Journal of Personality* 2014; **28**: 312-31.
42. QSR International Pty Ltd. NVivo qualitative data analysis Software Version 11.0. 2015.
43. Gale N, Heath, G., Cameron, E., Rashid, S., Redwood, S. Using the framework method for the analysis of qualitative data in multi-disciplinary health research. *BMC medical research methodology* 2013; **13**: 117.
44. Bazeley P. Qualitative Data Analysis, Practical Strategies. London: Sage; 2013.
45. Jennings H, Slade M, Bates P, Munday E, Toney R. Best practice framework for Patient and Public Involvement (PPI) in collaborative data analysis of qualitative mental health research: methodology development and refinement. *BMC Psychiatry* 2018; **18**: 213.
46. World Health Organization. Mental Health Action Plan 2013-2020. Geneva: WHO; 2013.
47. Slade M, Amering M, Oades L. Recovery: an international perspective. *Epidemiol Psichiatr Soc* 2008; **17**: 128-37.
48. HM Government. No health without mental health. Delivering better mental health outcomes for people of all ages. London: Department of Health; 2011.
49. Mental Health Commission of Canada. Changing directions, changing lives: The mental health strategy for Canada. Calgary: Mental Health Commission of Canada; 2012.
50. Department of Health. The Fifth National Mental Health and Suicide Prevention Plan. Canberra: Commonwealth of Australia; 2017.
51. Mental Health Commission. Strategic Plan 2016 - 2018. Dublin: Mental Health Commission; 2017.
52. Slade M, Amering M, Farkas M, et al. Uses and abuses of recovery: implementing recovery-oriented practices in mental health systems. *World Psychiatry* 2014; **13**: 12-20.
53. Tse S, Tsoi E, Hamilton B, et al. Uses of Strength-Based Interventions for people with serious mental illness: A critical review. *Int J Soc Psychiatry* 2016; **62**: 281-91.
54. Priebe S, Omer S, Giacco D, Slade M. Resource-oriented therapeutic models in psychiatry – A conceptual review. *Br J Psychiatry* 2014; **204**: 256-61.
55. Bird V, Le Boutillier C, Leamy M, et al. Assessing the strengths of mental health service users - systematic review. *Psychological Assessment* 2012; **24**: 1024-33.

For peer review only - http://bmjopen.bmj.com/site/about/guidelines.xhtml

56. Cook JA, Copeland ME, Jonikas JA, et al. Results of a randomized controlled trial of mental illness self-management using Wellness Recovery Action Planning. *Schizophr Bull* 2012; **38**: 881-91.
57. Slade M. Implementing shared decision making in routine mental health care. *World Psychiatry* 2017; **16**: 146-53.
58. Kirst M, Zerger S, Harris D, Plenert E, Stergiopoulos V. The promise of recovery: narratives of hope among homeless individuals with mental illness participating in a Housing First randomised controlled trial in Toronto, Canada. *BMJ Open* 2014; **4**: e004379.
59. Schrank B, Bird V, Rudnick A, Slade M. Determinants, self-management strategies and interventions for hope in people with mental disorders: systematic search and narrative review. *Soc Sci Med* 2012; **74**: 554-64.
60. Slade M, Oades L, Jarden A, editors. Wellbeing, Recovery and Mental Health. Cambridge: Cambridge University Press; 2017.
61. Schrank B, Riches S, Bird V, Murray J, Tylee A, Slade M. A conceptual framework for improving well-being in people with a diagnosis of psychosis. *Epidemiology and Psychiatric Sciences* 2014; **23**: 377-87.
62. Schrank B, Riches S, Coggins T, Rashid T, Tylee A, Slade M. WELLFOCUS PPT – modified Positive Psychotherapy to improve well-being in psychosis: study protocol for pilot randomised controlled trial. *Trials* 2014; **15**: 202.
63. Slade M, Brownell T, Rashid T, Schrank B. Positive Psychotherapy for Psychosis. Hove: Routledge; 2017.
64. Slade M. Mental illness and well-being: the central importance of positive psychology and recovery approaches. *BMC Health Services Research* 2010; **10**: 26.
65. Whitley R, Shepherd G, Slade M. Recovery Colleges as a mental health innovation. *World Psychiatry* in press.
66. Toney R, Knight J, Hamill K, et al. Development and evaluation of a Recovery College fidelity measure. *Canadian Journal of Psychiatry*: DOI:10.1177/0706743718815893.
67. Crowther A, Taylor A, Toney R, et al. The impact of Recovery Colleges on mental health staff, services and society. *Epidemiology and Psychiatric Sciences* in press.
68. Toney R, Elton D, Munday E, et al. Mechanisms of action and outcomes for students in Recovery Colleges. *Psychiatr Serv* 2018; **69**: 1222-9.
69. Puschner B. Peer support and global mental health. *Epidemiology and Psychiatric Sciences* 2018; **27**: 413-4.
70. Pitt V, Lowe D, Hill S, et al. Consumer-providers of care for adult clients of statutory mental health services. *Cochrane Database of Systematic Reviews* 2013; (3).
71. Funk M, Drew N. WHO QualityRights: transforming mental health services. *Lancet Psychiatry* 2017; **4**: 826-7.
72. United Nations General Assembly. Report of the Special Rapporteur on the right of everyone to the enjoyment of the highest attainable standard of physical and mental health. New York: Human Rights Council; 2017.
73. Sweeney A, Clement S, Filson B, Kennedy A. Trauma-informed mental healthcare in the UK: what is it and how can we further its development? *Mental Health Review Journal* 2016; **21**: 174-92.
74. Pietruch M, Jobson, L. Posttraumatic growth and recovery in people with first episode psychosis: an investigation into the role of self-disclosure. *Psychosis* 2012; **4**: 213-23.

For peer review only - http://bmjopen.bmj.com/site/about/guidelines.xhtml

For peer review only - http://bmjopen.bmj.com/site/about/guidelines.xhtml

Patient and Public Involvement statement

Patients were involved in acquisition of funding, both through a Lived Experience Advisory Panel informing the design and through involvement as applicants. Several interviewers and analysts had their own lived experience of mental ill-health and recovery, in addition to their professional training. The NEON Lived Experience Advisory Panel (LEAP) comprising ten members with lived experience of mental ill-health and recovery informed the ethics application, trained the interviewers, informed the topic guide and supported access to Group D. A LEAP member was involved in interpretation of the findings and as a co-author of this paper. The LEAP are leading the writing of a guide to sharing stories, which will be informed by this research.

Funding statement

This article is independent research funded by the National Institute for Health Research (NIHR) under its Programme Grants for Applied Research Programme (Narrative Experiences ONline (NEON) Programme, RP-PG-0615-20016). MS acknowledges the support of Center for Mental Health and Substance Abuse, University of South-Eastern Norway and the NIHR Nottingham Biomedical Research Centre (BRC). GT is supported by NIHR Collaboration for Leadership in Applied Health Research and Care South London (King's College London NHS Foundation Trust), Department of Health via the NIHR BRC and Dementia Unit (South London and Maudsley NHS Foundation Trust in partnership with King's College London and King's College Hospital NHS Foundation Trust), European Union Seventh Framework Programme (FP7/2007-2013, Emerald project), National Institute of Mental Health (National Institutes of Health R01MH100470, Cobalt study) and UK Medical Research Council (MR/S001255/1 Emilia; MR/R023697/1 Indigo Partnership) awards. The views expressed are those of the authors and not necessarily those of the NHS, the NIHR, the Department of Health and Social Care or other supporting funders.

Competing interests statement

None declared

Author statement

MS and LB made a substantial contribution to the conception or design of the work. MS, SRE, LB, JLB, DF, AH, GT, RM, KP, SP, AR, DR and ED contributed to the acquisition, analysis, or interpretation of data for the work. MS, SRE, LB, JLB, DF, AH, GT, RM, KP, SP, AR, DR and ED were involved in drafting the work or revising it critically for important intellectual content, and gave final approval of the version to be published. MS, SRE, LB, JLB, DF, AH, GT, RM, KP, SP, AR, DR and ED agree to be jointly accountable for all aspects of the work.

Data sharing statement

All data are included.

Licensing statement

I, the Submitting Author has the right to grant and does grant on behalf of all authors of the Work (as defined in the below author licence), an exclusive licence and/or a non-exclusive licence for contributions from authors who are: i) UK Crown employees; ii) where BMJ has agreed a CC-BY licence shall apply, and/or iii) in accordance with the terms applicable for US Federal Government officers or employees acting as part of their official duties; on a worldwide, perpetual, irrevocable, royalty-free basis to BMJ

Publishing Group Ltd ("BMJ") its licensees and where the relevant Journal is co-owned by BMJ to the co-owners of the Journal, to publish the Work in BMJ Open and any other BMJ products and to exploit all rights, as set out in our licence.

The Submitting Author accepts and understands that any supply made under these terms is made by BMJ to the Submitting Author unless you are acting as an employee on behalf of your employer or a postgraduate student of an affiliated institution which is paying any applicable article publishing charge ("APC") for Open Access articles. Where the Submitting Author wishes to make the Work available on an Open Access basis (and intends to pay the relevant APC), the terms of reuse of such Open Access shall be governed by a Creative Commons licence – details of these licences and which Creative Commons licence will apply to this Work are set out in our licence referred to above.

For peer review only - http://bmjopen.bmj.com/site/about/guidelines.xhtml

Table 1: Clinical and sociodemographic characteristics of participants (n=77)

Characteristic	Total	Group A (Outside the system)	Group B (BAME)	Group C (Under-served)	Group D (Peer)
n (%)	*77 (100)*	*21 (27)*	*21 (27)*	*19 (25)*	*16 (21)*
**Gender** n (%)					
Female	42 (55)	14 (67)	11 (53)	8 (42)	9 (56)
Male	30 (39)	6 (29)	9 (43)	9 (47)	6 (38)
Other / prefer not to say	5 (6)	1 (5)	1 (5)	2 (11)	1 (6)
**Ethnicity** n (%)					
White British	44 (57)	12 (57)	0 (0)	18 (95)	14 (88)
Black British	5 (6)	2 (10)	3 (14)	0 (0)	0 (0)
Black African / Caribbean	4 (5)	1 (5)	3 (14)	0 (0)	0 (0)
White Other	5 (6)	2 (10)	1 (5)	0 (0)	2 (13)
White and Black African / Caribbean	4 (5)	0 (0)	4 (19)	0 (0)	0 (0)
Asian / Mixed white Asian	4 (5)	0 (0)	4 (19)	0 (0)	0 (0)
Other	5 (6)	2 (10)	3 (14)	0 (0)	0 (0)
Prefer not to say	6 (8)	2 (10)	3 (14)	1 (5)	0 (0)
**Age** (years)					
18-25	4 (5)	0 (0)	0 (0)	3 (16)	1 (6)
25-34	16 (21)	3 (14)	6 (29)	4 (21)	3 (19)
35-44	16 (21)	5 (24)	4 (19)	4 (21)	3 (19)
45-54	30 (39)	8 (38)	9 (43)	6 (32)	7 (43)
55+	5 (6)	4 (19)	0 (0)	0 (0)	1 (6)
Prefer not to say	6 (8)	1 (5)	2 (10)	2 (11)	1 (6)
**Sexual orientation**					
Heterosexual	49 (64)	15 (71)	14 (67)	6 (32)	14 (88)
LGBT+	18 (23)	3 (14)	4 (19)	9 (47)	2 (13)
Prefer not to say	10 (13)	3 (14)	3 (14)	4 (21)	0 (0)
**Primary diagnosis**					
Schizophrenia or other psychosis	11 (14)	5 (24)	4 (19)	2 (11)	0 (0)

For peer review only - http://bmjopen.bmj.com/site/about/guidelines.xhtml

Bipolar disorder / cyclothymia	16 (21)	8 (38)	1 (5)	3 (16)	4 (25)
Mood disorder, e.g. anxiety, depression, dysthymia	15 (19)	1 (5)	4 (19)	4 (21)	6 (38)
Other, e.g. ADHD, personality disorder, substance abuse, autism	7 (9)	0 (0)	2 (10)	3 (16)	2 (13)
Prefer not to say	28 (36)	7 (33)	10 (48)	7 (37)	4 (25)

Table 2: Final conceptual framework for post-traumatic growth in psychosis and other severe mental health conditions

Type of growth	Definition of the positively perceived change
**1. Self-discovery**	**Having a fuller and deeper understanding of oneself**
1.1 Emotional life	Discovering or re-discovering how to access, accept and be mindful of inner emotional life and difficult feelings
1.2 Self-knowledge	Knowing oneself better, being more authentic and not being as shaped by the expectations of others
1.3 Self-acceptance	Grieving and letting go of the past, and developing self-compassion
1.4 Self-responsibility	Taking (back) responsibility for one's own life
**2. Sense of self**	**Development of a more positive sense of self, including integration and valuing of illness experiences**
2.1 Pride in self	Taking pride in oneself, including personal strengths and achievements
2.2 Integration of experiences	Illness experiences become an accepted part of one's sense of self
2.3 Valuing of experiences	Finding positives in the experience of illness
**3. Life perspective**	**New or renewed appreciation of or gratitude about aspects of life**
3.1 Appreciation of life	Appreciation for life and the importance of hopefulness
3.2 Appreciation of support	Gratitude for support received from services
3.3 Meaningful suffering	Gratitude that suffering was meaningful and not in vain
3.4 Survivor mission	New growth of political consciousness or use of illness experiences to benefit others
**4. Wellbeing**	**More active engagement in, and management of, one's own wellbeing and lifestyle**
4.1 Motivation	Increased determination to stay well, self-manage and not return to a bad situation
4.2 Being active	More engagement in the arts, music, sport, nature and learning
**5. Relationships**	**More actively choosing and valuing relationships with others**
5.1 Choosing relationships	Actively choosing relationships to continue, to re-start or to end
5.2 Valuing relationships	Placing more value on relationships with others
5.3 Empathy	Enhanced ability to empathise with others
**6 Spirituality**	**Deeper engagement with spirituality, religious and existential endeavours**
6.1 Spiritual awareness	Increased awareness of the presence of something greater than oneself making a positive contribution by providing meaning
6.2 Spiritual engagement	New or renewed engagement with spiritual or religious practices, helping with meaning-making and providing comfort and security

For peer review only - http://bmjopen.bmj.com/site/about/guidelines.xhtml

Online Supplement 1

Guidelines for promotional material

All promotional material produced to support recruitment in NEON Phase 1, for both activities 1 and 2, will incorporate the following:

1. A summary of the objectives of the project as a whole, e.g.
"The NEON Study looks at whether experiencing online stories of personal recovery ('recovery narratives') told by peers improves quality of life for people with psychosis."

2. A summary of the objectives of phase 1, e.g.
"The aim of phase one is to develop a theoretical understanding of how recovery narratives (personal stories of mental health problems and recovery) can be of benefit to other people with mental health problems."

3. A summary of the group which the promotional material is aiming to recruit, e.g.
"We are seeking to recruit 30 participants who are working in statutory or voluntary roles where having lived experience of mental health problems is a requirements, and where sharing personal experiences is a normal part of the work of the role"

4. A summary of what participation would require, e.g.
"Each participant would engage in a single one-hour interview, where they will be asked to talk about their experience of recovery".

5. The corporate identity of Nottinghamshire Healthcare NHS Foundation Trust (NHCT), as the project sponsor (e.g. see header of this document)

6. If this piece of recruitment is located in a site other than NHCT, then the corporate identity of the site where it is being conducted.
This will be either South London and Maudsley NHS Foundation Trust (SLAM) or East London NHS Foundation Trust (ELFT).

7. A mechanism to allow people to find out more information, e.g.
- An email address or telephone number
- An information page on the project website

8. Information about the funder and mechanism, e.g.
"This project has been funded by the National Institute of Health Research, as a Programme Grant for Applied Research (PGfAR)"

9. Information about approvals that have been received, e.g.
"This study has been reviewed and given favourable opinion by INSERT REC INFO and CONTACT INFO. The project has also been reviewed by the Health Research Authority, and by the Research and Innovation Team at Nottingham Healthcare NHS Foundation Trust, who are sponsoring the project."

For peer review only

Online Supplement 2

Preliminary coding framework for post-traumatic growth in psychosis and other severe mental health conditions

#	Code	Definition	Examples
1	Strengths	New or improved use of personal strengths or resources	More resilience, confidence, self-efficacy, skills at navigating life challenges, inner strength, determination
2	Relationships	Improved relationships with others	Better connection with friends or family, new meaningful relationships
3	Purpose	New possibilities and purpose in life	Fresh directions, e.g. career, leisure pursuits; finding a place or role in society
4	Life appreciation	Greater or altered appreciation of life	Finding new meaning in experiences, seizing opportunities, finding new life purpose or direction
5	Spirituality	More engagement with spirituality or existential questions	New spiritual practice, (re-)connecting with an organised religion, deeper life meaning
6	Character	Developing positive character traits	More creativity, compassion for others
7	Lifestyle	Making positive lifestyle changes	Better sleep, more activity, healthier relationship with food / drink, developing new interests, choosing healthier relationships
8	Identity integration	Integration of experience of psychosis into identity	Psychosis experiences as shaping who the person is now, psychosis being part but not all of identity, more insight or clarity
9	Self-acceptance	More self-acceptance and awareness	More self-compassion, positive sense of self
10	Other	Other forms of post-traumatic growth not coded above	

For peer review only

Online Supplement 3

NB The interview was in two parts. In Part 1 the participant was asked to tell their story of recovery. In Part 2, follow-up questions were asked about the impact of their stories on others. The topic guide for Part 1 did not change, and only data from Part 1 (i.e. the participant's open narrative) are reported in the current study.

Interview topic guide

START OF TOPIC GUIDE

Preparation

Before the participant arrives, allocate a unique identifier (UID) to the participant, and write it into a blank Informed Consent Form.

Introduction

- Introduce the interviewer facilitator
- Ensure the participant has read the information sheet and understands that participation is voluntary and that they are free to withdraw at any time
- Explain the aims and purpose of the activity and give a brief description of the interview structure. Tell the participant they can decide whether their story is used in the later part of the NEON study, emphasising that this may mean other people beyond the study team may see it.
- Provide an opportunity for participant to ask any questions, and then obtain written consent through the Informed Consent Form
- Describe digital recording of the interview. Clarify whether the participant wants to be video-recorded or audio-recorded.
- Turn on the recorder. Read out the UID so that it is recorded.

Questions

Part 1

Ask the participant to describe their own story of recovery. Do not use language (e.g. 'psychosis') which might be leading – refer to e.g. 'mental health difficulties'. Give them plenty of time, listen carefully. Reply if asked (e.g. "Is this okay?" – "Yes, you're doing great") and use minimal prompts if needed (e.g. "Do go on") but try to let the participant tell their story in their own words.

Part 2

Follow up with questions about the narrative, e.g.

1. Who have you shared your story with, and why?
2. What was the impact on the recipient and on you? If there was a particularly powerful part of their narrative, ask specifically about the impact of that part of their story.
4. Do you sometimes hold back some aspects of your story? If so, how do you decide what and when to hold back?

For peer review only

3. Has anyone else shared a recovery narrative with you? What parts of someone else's story made an impact? Why do you think that part made an impact?
4. How has the way you have told your story changed over time? Why do you think this is?

If the participant becomes distressed during the interview, ask if they would like to take a break or stop. If the participant reveals information which is of concern and may need reporting i.e. potential risks to another person or to themselves, or criminal behaviour, then continue the interview if you feel comfortable to, but discuss these with the PI at the earliest opportunity and where appropriate report accordingly.

End of interview
Explain the interview is now over and ask if the participant has any final questions. Give information about timeline for the study and how publications can be accessed. Thank the participant for their participation.

END OF TOPIC GUIDE

For peer review only

Online Supplement 4

Interim coding framework for post-traumatic growth in psychosis and other severe mental health conditions

#	Code	Definition	Examples
1	Strengths	New or improved recognition or use of personal strengths or resources	More resilience, confidence, self-efficacy, skills at navigating life challenges, inner strength, determination, positive character traits
2	Relationships	Improved relationships with others	Better connection with friends or family, new meaningful relationships, moving away from unhealthy relationships
3	Purposefulness	New possibilities and purpose in life	Fresh directions, e.g. career, leisure pursuits; finding a place or role in society, social or political activism.
4	Life appreciation	Greater or altered appreciation of or reflection on life	Fresh perspectives, new meaning-making framework. Finding new meaning in experiences, seizing opportunities, finding new life purpose or direction, new appreciation for informal or formal learning, new or different education or career development
5	Spirituality	More engagement with spirituality or existential questions	New spiritual practice, (re-)connecting with an organised religion, deeper life meaning
6	Self-discovery	Having a changed, fuller and deeper understanding of oneself and how to live in the world	More creativity, compassion for others, increased empathy for others, new skills
7	Lifestyle	Making positive lifestyle changes to maintain wellbeing and support self-care	Better sleep, more activity, healthier relationship with food / drink, developing new interests, choosing healthier relationships, changing social environment
8	Identity integration	Integration and sense-making of experience of mental health issues into identity	Mental health experiences as shaping who the person is now, psychosis or other mental health experiences being part but not all of identity, more insight or clarity, re-framing experiences, choosing other explanatory frameworks to make sense of experiences
9	Self-acceptance	More self-acceptance and self-compassion	More self-compassion, positive sense of self, less internalised stigma
10	Other	Other forms of post-traumatic growth not coded above	

For peer review only

Online Supplement 5

Final conceptual framework for post-traumatic growth in psychosis and other severe mental health conditions

**Type of growth**	**Definition of the positively perceived change**	**Illustrative quotes**
**1. Self-discovery**	**Having a fuller and deeper understanding of oneself**	
1.1 Emotional life	Discovering or re-discovering how to access, accept and be mindful of inner emotional life and difficult feelings	But what I will say is that, er, I found that the, my, what was going on was an internal not an external thing. Because before that I, I've moved about nine times, I'd move, I'd just move somewhere else and thought it's going to be different now. At one point I moved to the States, you know? And I'd be in the same situation in the States that I'd be in England. (B04)<br><br>There was just a point where I was just like I forgot how to feel, I forgot how to be hungry, I forgot how to feel tired, I forgot how to be exhausted, I forgot how to feel fear and then I realised oh my fuck I need to feel fear, you know, I need these things, these things are really fucking important, you know that a human being needs fear, we need tiredness, we need hunger…I moved to Hong Kong and it found me again and I realised it wasn't really my situation, it was me, it was something inside me that was drawing this energy towards me and there I, unless I changed the noise inside, the noise outside is never going to go away. (B25)<br><br>And I think that is the key to everything isn't it, accessing your emotions, not running away from them, which a lot of people in mental suffering with mental health are trying to do because it is overwhelming and painful. So it is about yeah feeling and not feeling somebody else's or not, following somebody else and not being scared to be who you are and be different I think. (C19)
1.2 Self-	Knowing oneself	I feel like I know myself quite well, you know I can heal myself. And uh, these are things

For peer review only

knowledge	better, being more authentic and not being as shaped by the expectations of others	that people actually take for granted but took me a very long time to connect to how are you feeling you know and what do you want right now? (A08)<br><br>I think you find out who you are and sort of how you have to live to keep yourself sane in a way. (A14)<br><br>When I discovered that freedom, that I didn't have to join the rat-race of having a car, wife and three kids, you know, having a job, that was quite liberating… I was constrained by what people wanted me to do, instead of what I wanted to do. (B18)<br><br>People or old friends that would say…you've got to stop being a bit like this, actually I would take that and think to myself am I? Am I a bit like that? And question what they've said and then if I thought well no actually I'm not like that anymore, you know, then I've turned around and said well actually, you know, that's wrong, you know, because that's, that's, that's not me and you can't tell me I am something if that's not what I am. You know, well you can but you know I'm not gonna listen to you. And uh you know by, by questioning uh what people was saying, uh sort of, uh, removed that vulnerability I think, and um, by removing vulnerability has allowed me to feel more in control of myself and um, who I am. (C03)
1.3 Self-acceptance	Grieving and letting go of the past, and developing self-compassion	I grieve all the hopes that are not there, you know things that haven't worked out, and I try to accept what is there and say well this is it, this is the thing that is real, maybe the other things were a little fantastical… And so I still talk to myself a lot but it's not now, it's, it's, it's me comforting myself and actually communicating with me, you know, just sort of, um it's that thing of having learned to love myself. (A08)<br><br>I started going to loads of classical music concerts which I'd really shunned because I felt like I'd really failed as I'm not playing the violin anymore, I should be, I've really failed, I'm not good as the other people who have made it their profession. Suddenly I was like, God that doesn't matter, that is just so irrelevant, like classical music and music generally is so beautiful I just want to go and listen to it. (A10)<br><br>It's all about self-accepting, getting to know me and it helped. (A17)

For peer review only

		I feel like I'm at peace with a lot of the, erm, depression that I felt over things that have happened in the past. (B01)<br><br>The key word is accepting the situation that I was in, um, and being honest with myself. (C03)
1.4 Self-responsibility	Taking (back) responsibility for one's own life	Yeah, because in my own mindset and recovery…I had to become interested in the problem because prior to that my outlook on recovery in mental health was based on seeing a Consultant Psychiatrist and they will have the answer because that is the predominant feeling, patient/doctor relationship, you see a doctor, you trust what they say and then I had to become interested after I realised that the doctors can't fix me. So when I became interested I became open to learning. (A21)<br><br>The one thing he did when he came, when he saw, he said, 'I'm not going to help you do one thing in this flat unless you start doing it for yourself. If you make a start yourself, I will help you. If you don't do anything, I'm gone. And that hit me like a, er, thunderbolt, knowing that, knowing I, that if I'm not going to help myself, no one else will help me. And that was the beginning, really, of my recovery. (B02)<br><br>Everything I do is like an attempt to rebuild my life but it's taken a long time. (B05)<br><br>Maybe sometimes we need to go through that process, in order to think, I don't need to go back there, to where I was ten years ago, thinking about that experience anymore. But you can think about it, but have a different sort of attitude to it. (B06)<br><br>I tend to be living the moment, that's part of my new self, this post-near death experience self is to really be living in the moment as much as possible, not worry about the future or churn over the past. (D04)<br><br>This was like taking a step back and looking at almost re-engineering life to take into account self-care, self-preservation and also building myself up rather than saying rotten stuff about myself and focusing on what I couldn't do and what I should do and should be

For peer review only

		able to do and all of that. (D08)
**2. Sense of self**	**Development of a more positive sense of self, including integration and valuing of illness experiences**	
2.1 Pride in self	Taking pride in oneself, including personal strengths and achievements	I feel quite proud of my stability. (A01)<br><br>I'm proud of that and that makes me feel better and enables me to operate…I've worked on myself throughout my life and I'm quite proud of that. (A12)<br><br>I believe in my self-worth these days…I must pat myself on the back. (A15)<br><br>Doing well, embracing who you are, loving who you are. Because I went through a long period of neglecting myself, my identity, my race, my culture. (B08)<br><br>I think that's something I'm pretty proud of actually, that I just take people as they are. (B21)<br><br>So yeah, I'm proud of where I've come, do you know what I mean, how far I've come, and that's what help, holds me from stopping and falling apart completely when I think about my daughter, and I get real mad sometimes…That knowing what I've got, and how I've achieved, and I don't want to lose it, cos when she comes to find me I want her to see that I've got these things, and that I'm not a complete arsehole living in a hostel, know what I mean? (C10)
2.2 Integration of experiences	Illness experiences become an accepted part of one's sense of self	That stuff has happened in my life and that is just how it has turned out. Um yeah, that is unfortunate but it is not going to define everything about me. (A07)<br><br>If there was a big red button on the table and somebody said would you like to switch off your diagnosis I would say no because I have accepted it now. Although it was horrid, it

For peer review only

		was horrible, I can't take anything away from that, but I have accepted that it has become part of my personality and I am happy with that and that's me. (A21)<br><br>I think for a long time I was just existing with the mental health, getting to know, getting to understand it…Because there's very much negativity around it and I felt ashamed. (B06)<br><br>You don't choose the issues that you've got but you can, you can make a choice to change. (C04)<br><br>I am who I am because of what happened. (D04)<br><br>The more I was able to make sense through telling my story of what had happened to me, it then started to have a meaning. (D11)
2.3 Valuing of experiences	Finding positives in the experience of illness	I've had all these amazing experiences and you are just saying it's illness, I just didn't believe it…I feel that it's important and the messages I've got from being in an alternative reality have been extremely important. (A10)<br><br>What they were calling symptoms that must be eradicated, were actually part of me and so I looked behind that and said that is where, that is where my creativity comes from. This is where, this is how I am able to take a photograph, this is how I am able, when I am sitting at a piano to think of a tune. (A19)<br><br>Whereas before I felt ashamed to have these mental health issues, I now feel really proud in a way because I know that I can use it for good and I can make a difference in my community. (B01)<br><br>There is a liberation that comes through all of this, a personal kind of yeah, not sure it's full blown self-actualisation, but it's somewhere up close to the top of the pyramid. (D04)<br><br>I am still me but I am a different me and I am stronger. (D05)<br><br>I kind of thought that that was the best I could ever hope for and that was fine because I

For peer review only

		kind of really internalized my label of I am an anorexic, I am a you know a broken person who needs a lot of support and actually it was seeing the people who were talking about their lived experience with lots and lots of confidence and taking lots of responsibility and um being empowered to, to self-manage and use their difficulties in a really positive way which kind of opened my eyes to like gosh maybe I am more than my label, maybe I am, maybe I could do more than just function or survive, maybe I could thrive. (D014)
**3. Life perspective**	**New or renewed appreciation of or gratitude about aspects of life**	
3.1 Appreciation of life	Appreciation for life and the importance of hopefulness	I suppose I've become one of those ridiculously, someone I thought I'd never be, I'm becoming one of those ridiculously ever hopeful, ever optimistic people who say there is hope, my life is a life that is about hope, I hope. (A08)<br><br>I have kind of realised that I am not in a position to dictate the terms of life: “it should be like this and I expect this and why is it not like this”. Just, I am alive, I appreciate that I am alive and that I have got so many hours each day to gain something from, some joy or some inspiration or blessings and gifts everywhere that I can and the number one thing is to just keep going, I am a mother and I have to keep going. (C19)
3.2 Appreciation of support	Gratitude for support received from services	So it [organisation] completely changed my view of life. And it's a, it's a roller coaster, it's not like every day's joyous, things would dip, challenges still come in but I deal with them completely differently. Yeah it's like I've found happiness that I never knew existed. (A11)<br><br>Rehab and coming to <service name 1> changed my life, it's like <worker> got me on a college course, it has been absolutely wonderful, it really has, I did a theatre group … and through the rehab I got told about [inaudible] and I came down here <service name 2> and this place is amazing, the things they do here for anybody involved in sex work is absolutely amazing. (C18)
3.3 Meaningful suffering	Gratitude that suffering was meaningful and not in vain	Wow I see where I am and I go back to then and I just think 'you didn't go through that in vain'. (A17)<br><br>So I think I'm, I suppose I am grateful, for want of another way of putting it, that I have

		lived the life I have, I have had these experiences. (D04)<br><br>And it's okay, because I wouldn't be where I was, had I not had the experiences that I've had. I don't know what sort of person I'd be, but I certainly wouldn't be where I, doing what I'm doing now. (D12)
3.4 Survivor mission	New growth of political consciousness or use of illness experiences to benefit others	<Organisation> is about re-framing mental illness as a possible catalyst for possible transformation, so and the aim is to give hope to as many people as possible through the power of story-sharing, so I'm really grateful and passionate about this. (A11)<br><br>I just see myself as hopefully being a beacon to others who are, you know, struggling, and others who are finding things difficult. And they can, you know, refer to me [inaudible] support person and they can look at me and think 'He can make it through all the things that happened to him, then I can make it too'. (B02)<br><br>I think it's really important to give back to society. And I think that's what I'm doing through my work. This new chapter I'm going to start, with women now. (B06)<br><br>And so, you know, I felt like I was becoming something. Erm, I could identify with more, kind of, politicised…the personal was political and I was beginning to become aware of that on a deeper level. So, I thought right, that's it and, I'm going to set up my own thing. (B09)<br><br>And then as I came back out it was just like no, I wanted to help, I wanted to do something, I wanted to use this story, I wanted to actually like you know... I wanted, that's when I started studying psychology and then I applied to go study in <place>…what's helped me recover was I find life, you know, I've got stuff I want to do, I want to help people, I want to, I want to do psychology. (B25)<br><br>I've been looking forward to sharing my experience because um, by people sharing theirs has really helped me and you know, and that makes it feel like talking about it is a really worthwhile thing to do, because its gonna allow somebody else to grow, or just take a little bit from it that you know, it might benefit them or it might not, maybe they

		need to hear a different story but, you know, somebody might, you know, might hear it and think well I'm really pleased that I've heard that because that's, that's gonna help me. (C03)<br><br>I guess in my recovery I tried to be that person for other people, the thing that I didn't have. (C04)<br><br>And my journey through prostitution, uh so I can talk to people about it now. And that's what I want to do, I want to go on and help people if I can that have been through the same thing. (C18)
**4. Wellbeing**	**More active engagement in, and management of, one's own wellbeing and lifestyle**	
4.1 Motivation	Increased determined to stay well, self-manage and not return to a bad situation	The violence started again and I had two broken jaws, a broken nose, scars on my head and scars on my body, but the biggest scar was in my heart and in my mind. I ended up... being put into a secure unit this time and I was meant to be there for three months. It ended up being two weeks, becoming a voluntary patient for a year. Touch wood and whistle, I swore from that day on, no man, money, love or beast would ever put me back into that situation again and I have stuck to it. (A15)<br><br>I started to take my medication, I got rid of this awful man in my life who was my daughter's father. Which was very liberating and, suddenly had an epiphany through all the craziness and thought, I don't want this in my life anymore. If I get rid of certain things it will decrease some of the stress. (B06)<br><br>I have been that low. And that ill, I have been there and I have seen the light and…I have come back from it. I have actually come out of it, I've been there, right there to the lowest of the low. And pulled myself back from it. And that's difficult to do. (C12)

For peer review only

		When I came out of hospital I was like "I can't go back there again so I've got to sort myself out and I've got to build a better relationship with me and the girls", you know, they'd been through so much. (D05)<br><br>Rather than just aimlessly sort of treading water and doing things that I felt I was sort of having to do or other people were you know were saying I should do you know sort of planning about things that actually I enjoyed. (D13)
4.2 Being active	More engagement in the arts, music, sport, nature and learning	I think the art has given me great kind of, great kind of structure. (A01)<br><br>I didn't go into that dangerous space again but I did meet a lot of like-minded people and I played my violin a lot and I decided I wanted to do something with my violin…I would get like a kind of vision of what my purpose was, what my true purpose was in my life and, it was like you know, playing the violin, teaching the violin, it was, which I hadn't been doing because I was working in admin but it just felt really strongly, that is what, it wasn't to do with competition it just felt like, that's the right direction for me to go in and I need to go more and more in that direction, it felt like, like it was connecting me to my soul purpose somehow. (A10)<br><br>I am someone that struggles with relaxing. So, um, gym, I find that I am someone who has to go to the gym, I have to exercise, um and as ridiculous as it sounds, eating as well. You know like I am someone that needs to treat myself good. In order to be good in myself. (A14)<br><br>So that was quite liberating and I didn't think that it would help my mental health without even realising, but I think it did, I did become a bit obsessed with jogging because it was something, I guess, a bit of a break from my children and the monotony of life and the difficult relationship I was in, and the difficulties I had. (B06)<br><br>And erm, and I believe that I need to give more time, a bit more time to, to interests. You know, like, er, I love nature. Absolutely adore nature, it's one of the places I've always felt safe, I could go and sleep in forests, more than I could go and sleep in someone's house. (B09)

For peer review only

		I've been reading quite a lot and educating myself on schizo-affective disorder, plus all the things I've learned about meditation and that kind of thing I practice daily, and I've just found learning to be so rewarding and things, learning through recovery college as well, even though they do suggest reading up on your condition and that kind of thing. So I've just found learning to be so therapeutic and rewarding that I feel like I am at a point now where I can actually study and put my mind towards…doing something worthwhile. (B10)<br><br>I play a lot of music, music is like my, it's my salvation. (B25)<br><br>I could see my body changing and I felt stronger and I felt better and I stopped drinking for a month and I just felt amazing! (D05)<br><br>It was also about sort of about finding activities that I enjoyed much more, rather than just aimlessly sort of treading water and doing things that I felt I was sort of having to do or other people were you know were saying I should do, you know, sort of planning about things that actually I enjoyed… One of the things I did was I joined the choir and you know it's one of [inaudible] gospel choir in South London for about, ever since I got there really and it's a very, very supportive environment, so that's been really critical and I've been able to sort of go there when I've been in every kind of mood state possible. (D13)
**5. Relationships**	**More actively choosing and valuing relationships with others**	
5.1 Choosing relationships	Actively choosing relationships to continue, to re-start or to end	He (counsellor) used a waiter analogy where the waiter is trying to please the clients but then more and more people come in and he is still trying to please them all but the quality of the food comes down and then people start complaining and then it is mad, so basically just concentrate on what you can do and don't try to please everybody…I also now try to mix with people who are just happy and positive and try to stay away from

For peer review only

		people who aren't. (A09)<br><br>But you need the right quality of people around you, network, for you to be able to stay in your mental health, going forward. (B02)<br><br>I got rid of this awful man in my life. (B06)<br><br>I needed to go back to a couple of my old primary school friends' houses and ask for their forgiveness for something. (B07)<br><br>I'd also come to realise before then that the relationship I was in was not quite what it was as he told me lots of things that were untrue that I discovered so I ended that as well and that was having a clean slate but it took me a long time to get literally over it. (B17)
5.2 Valuing relationships	Placing more value on relationships with others	My children they come at the weekend I give them 100%. I give them 100% because I want to be giving them 100%. (A01)<br><br>But then in another sense it's brought the incredible closeness with people with mental health with my, with my immediate family and friends so it has different angles. (A03)<br><br>It's been a process of learning that I needed, I need desperately, I desperately needed family, you know, people I feel safe with, to be myself. And I needed people to help me normalise my feelings rather than be freaked out and hiding and terrified of expressing. (B09)<br><br>Now I do feel a lot more stable than I did back then, and then when I go to these kinds of experiences you know, I've started to like, connections have become my thing because connections are what saved me from like, connections with people, a spiritual connection and this connection between people, there's interconnection between people is essentially what saves people…That power of coming together is so powerful, that is worth fighting for, worth dying for, it's worth me giving my time for. (B25)<br><br>Having the friendship, the respect that built up over that in learning from each other, you

For peer review only

		know educated, talented, intelligent people. (C04)
5.3 Empathy	Enhanced ability to empathise with others	I can empathise and I do empathise and I use empathy in my work a lot. (A12)<br><br>So my purpose really is young people and even when I see the destruction that young people are going through I never blame them, I said it stems from somewhere…I knew what I went through as a young girl. I could have been a prostitute, I could have been on drugs, I didn't. Do you see what I'm saying? So therefore it's deep. (A17)<br><br>You have understanding, empathy…You really empathise. (B15)
**6. Spirituality**	**Deeper engagement with spirituality, religious and existential endeavours**	
6.1 Spiritual awareness	Increased awareness of the presence of something greater than oneself making a positive contribution by providing meaning	Although the experiences were horrendous, and they were horrendous, there's no other way of describing the agony, it has been necessary because I couldn't do it on my own, so it still feels, and maybe this is just you know the spiritual aspect of my life but it still feels as though I do have like something watches over me and is helping me um and I survived. (A08)<br><br>I kind of relived stuff and at the same time just physically I wanted to let go of loads of things, I just kind of clutter cleared my whole house, really cleaned it, I got rid of loads of bits and pieces that I didn't need any more and as I went through that whole process it was like this massive opening, just kind of spiritual opening again…I felt very very free, I felt like I was being guided with everything, I felt very, very spiritual. Um lots of things happened that were very healing I suppose, played a lot of music…when I relived it and the emotion that was associated my voice really did, like it changed, it kind of opened out and it became much more free and liberated and um at the same time I felt like I went into a completely different reality, it was really incredible, it was like intensely spiritual and a feeling that everything was connected and it was though lots of chakras opened or something happened in my body and my mind. (A10)

For peer review only

		Now I'm sort of growing older I know they're spirit animals, I still have them and so it's the wolves that are the most powerful so I do feel protected by those. Um and the other one is [inaudible] spiritual as well I always used to feel some [inaudible] egocentric, sort of like, wounds, [inaudible] kind of reaching out to people because I've always felt like I'm [inaudible] ever since I was really little which has why I have gone into mental health, to help people. (A18)<br><br>But I think the longer deal is this, this, internal shift, they call it a psychic shift, an internal psychic shift, which is more about that spiritual experience and I don't know anything else that, that would work with me, do you know what I mean. And so, erm, since about 2009, that's what I've been kind of exploring in a, in a sort of ad hoc way. (B04)<br><br>But when you kind of realise that there is something bigger than you, you start to realise don't hurt yourself, have more respect for yourself, and you also don't harm others in a sense, not but everything falls into place. The world has meaning, has greater meaning. (C19)
6.2 Spiritual engagement	New or renewed engagement with spiritual or religious practices, helping with meaning-making and providing comfort and security	Like wow so I ran over to them and they kind of just guided me so I was just being guided by all of the spirits and stuff and they guided me up the hill into the woods. (A02)<br><br>…was actually, uh, around all of this I was sort of meditating and looking at the more spiritual aspects of my life and you know, just looking for, I was looking and seeking that help and I was, I was meditating. It, it, it brought a lot of pain but I was looking at that. (A08)<br><br>I became very um sort of what's the word, like spiritual as well. I found the church very comforting and kind of a place I would be, to trust and yeah, it just gave a sense of security for some reason. (A14)<br><br>So, you know, if it wasn't, if it wasn't for yoga, my Buddhist practice and community, who get, who don't, who, who see that there's something valuable from raw emotion, that

		there's energy in raw emotion…My understanding of Buddhism had, had grown. Erm, I'd been introduced to it, to the practice of chanting [inaudible], to reveal your own courage and compassion and wisdom and create, create more humane connections with people. And, I thought it was amazing energy to, to nourish, 'cos it wasn't based on any gods or idols or who was, you know, who was, who was cool and who wasn't. It's universal. It's a universal truth….the long and short of it, Buddhist practice helped [laughs]. More harmonious with myself and other people. (B09).<br><br>Every morning I put my hand on my heart and I pray and I meditate and I go into that space where it's myself…Now I feel like now I do feel a lot more stable than I did back then and then when I go to these kinds of experiences you know, I've started to like connections have become my thing because connections are what saved me from like, connections with people, a spiritual connection and this connection between people, there's interconnection between people is essentially what saves people. (B25)<br><br>I was still a Quaker at this point so that was really helpful, so I would go each week to have silent worship and that was really good, I saw it as an opportunity to kind of pause and have my brain calm down and yeah that was really lovely. (C15)<br><br>…think also a lot, mental health is to do with emotion, for me I can only find the emotional vocabulary within church. And many things that we are struggling with are deep spiritual psychic rooms and you can't find that power and that passion in a, in anything other than a spiritual context… So for me recovery is about spirituality, it is about God, that is me and that's what works for me, it might not be the same for everyone…just pray, pray. (laughs) It works, do it, don't be scared. (C19)

SRQR Checklist

No.	Topic	Item	Location in text
	Title and abstract		
S1	Title	Concise description of the nature and topic of the study Identifying the study as qualitative or indicating the approach (e.g., ethnography, grounded theory) or data collection methods (e.g., interview, focus group) is recommended	Title
S2	Abstract	Summary of key elements of the study using the abstract format of the intended publication; typically includes background, purpose, methods, results, and conclusions	Abstract
	Introduction		
S3	Problem formulation	Description and significance of the problem/phenomenon studied; review of relevant theory and empirical work; problem statement	Introduction
S4	Purpose or research question	Purpose of the study and specific objectives or questions	Introduction last paragraph
	Methods		
S5	Qualitative approach and research paradigm	Qualitative approach (e.g., ethnography, grounded theory, case study, phenomenology, narrative research) and guiding theory if appropriate; identifying the research paradigm (e.g., postpositivist, constructivist/interpretivist) is also recommended	Methods Procedures paragraph 2
S6	Researcher characteristics and reflexivity	Researchers' characteristics that may influence the research, including personal attributes, qualifications / experience, relationship with participants, assumptions, and/or presuppositions; potential or actual interaction between researchers' characteristics and the research questions, approach, methods, results, and/or transferability	Methods Procedures and Methods Analysis
S7	Context	Setting/site and salient	Methods Setting

For peer review only

		contextual factors	
S8	Sampling strategy	How and why research participants, documents, or events were selected; criteria for deciding when no further sampling was necessary (e.g., sampling saturation); rationale	Methods Setting
S9	Ethical issues pertaining to human subjects	Documentation of approval by an appropriate ethics review board and participant consent, or explanation for lack thereof; other confidentiality and data security issues	Methods paragraph 1
S10	Data collection methods	Types of data collected; details of data collection procedures including (as appropriate) start and stop dates of data collection and analysis, iterative process, triangulation of sources/methods, and modification of procedures in response to evolving study findings	Methods Procedures and Online Supplement 2
S11	Data collection instruments and technologies	Description of instruments (e.g., interview guides, questionnaires) and devices (e.g., audio recorders) used for data collection; if/how the instrument(s) changed over the course of the study	Online Supplement 2
S12	Units of study	Number and relevant characteristics of participants, documents, or events included in the study; level of participation (could be reported in results)	Table 1
S13	Data processing	Methods for processing data prior to and during analysis, including transcription, data entry, data management and security, verification of data integrity, data coding, and anonymization/deidentification of excerpts	Methods Procedures last paragraph
S14	Data analysis	Process by which inferences, themes, etc., were identified and developed, including the researchers involved in data analysis; usually references a specific paradigm or approach; rationale	Methods Analysis and Online Supplement 1 and 3

For peer review only

S15	Techniques to enhance trustworthiness	Techniques to enhance trustworthiness and credibility of data analysis (e.g., member checking, audit trail, triangulation); rationale	Methods Analysis
	Results/findings		
S16	Synthesis and interpretation	Main findings (e.g., interpretations, inferences, and themes); might include development of a theory or model, or integration with prior research or theory	Results Major themes
S17	Links to empirical data	Evidence (e.g., quotes, field notes, text excerpts, photographs) to substantiate analytic findings	Results Category descriptions and Online Supplement 4
	Discussion		
S18	Integration with prior work, implications, transferability, and contribution(s) to the field	Short summary of main findings; explanation of how findings and conclusions connect to, support, elaborate on, or challenge conclusions of earlier scholarship; discussion of scope of application/generalizability; identification of unique contribution(s) to scholarship in a discipline or field	Discussion
S19	Limitations	Trustworthiness and limitations of findings	Discussion paragraph 2
	Other		
S20	Conflicts of interest	Potential sources of influence or perceived influence on study conduct and conclusions; how these were managed	Competing Interests statement
S21	Funding	Sources of funding and other support; role of funders in data collection, interpretation, and reporting	Funding statement

For peer review only
